# A Broad Survey of Gene Body and Repeat Methylation in Cnidaria Reveals a Complex Evolutionary History

**DOI:** 10.1093/gbe/evab284

**Published:** 2022-02-01

**Authors:** Xinhui Zhang, David Jacobs

**Affiliations:** Department of Ecology and Evolutionary Biology, University of California, Los Angeles, USA

**Keywords:** Cnidaria, DNA methylation, CpG_o/e_, parasitism, gene bodies, repetitive elements

## Abstract

DNA methylation, an important component of eukaryotic epigenetics, varies in pattern and function across Metazoa. Notably, bilaterian vertebrates and invertebrates differ dramatically in gene body methylation (GbM). Using the frequency of cytosine-phospho-guanines (CpGs), which are lost through mutation when methylated, we report the first broad survey of DNA methylation in Cnidaria, the ancient sister group to Bilateria. We find that: 1) GbM differentially relates to expression categories as it does in most bilaterian invertebrates, but distributions of GbM are less discretely bimodal. 2) Cnidarians generally have lower CpG frequencies on gene bodies than bilaterian invertebrates potentially suggesting a compensatory mechanism to replace CpG lost to mutation in Bilateria that is lacking in Cnidaria. 3) GbM patterns show some consistency within taxonomic groups such as the Scleractinian corals; however, GbM patterns variation across a range of taxonomic ranks in Cnidaria suggests active evolutionary change in GbM within Cnidaria. 4) Some but not all GbM variation is associated with life history change and genome expansion, whereas GbM loss is evident in endoparasitic cnidarians. 5) Cnidarian repetitive elements are less methylated than gene bodies, and methylation of both correlate with genome repeat content. 6) These observations reinforce claims that GbM evolved in stem Metazoa. Thus, this work supports overlap between DNA methylation processes in Cnidaria and Bilateria, provides a framework to compare methylation within and between Cnidaria and Bilateria, and demonstrates the previously unknown rapid evolution of cnidarian methylation.

Significance

DNA methylation shows dramatically different patterns in bilaterian vertebrates and invertebrates, yet the origin of these differences is unclear. We present the first comprehensive survey of DNA methylation in Cnidaria, the sister group to Bilateria, and show that gene body methylation is likely the ancestral state for Eumetazoa and that several factors likely contribute to the complex evolutionary history of DNA methylation in Cnidaria. This work shows that DNA methylation is highly conserved in Cnidaria, and provides an important piece of the puzzle of the evolution of DNA methylation in animals.

## Introduction

In Metazoa, cytosines in cytosine-phospho-guanine dinucleotides (CpG sites) are the predominant target of methylation. However, the overall level and pattern of such methylation varies greatly across Metazoa. Methylated cytosine is hypermutable converting to thymine at a high rate. In this work we use the resulting depletion of CpG sites as a proxy for DNA methylation, examining 76 species across Cnidaria. Although bisulfite sequencing remains the gold standard in DNA methylation research, it would require high-cost de novo sequencing in these 76 taxa to achieve this wide range of sampling (Dimond and Roberts 2016; Aliaga et al. 2019). Thus, our application of this proxy provides a critical avenue forward for evolutionary study, as methylation sequencing outside of vertebrates is limited and CpG depletion can be measured using genomic and transcriptomic data accumulated for other purposes.

Cnidaria, the sister group of Bilateria, contains a suite of ancient taxa with substantial variation in life history, symbiosis, coloniality, and parasitism. Thus, Cnidaria provides an interesting system to understand the evolution of DNA methylation across the Metazoa. For the rest of the introduction, we will review: 1) the patterns of DNA methylation in different Metazoan taxa, 2) the two main gene families involved in DNA methylation machinery in Metazoa—DNA methyltransferases (DNMTs) and methyl-CpG-binding domain proteins (MBDs), and 3) the measurement of CpG depletion.

**Fig. 1.**
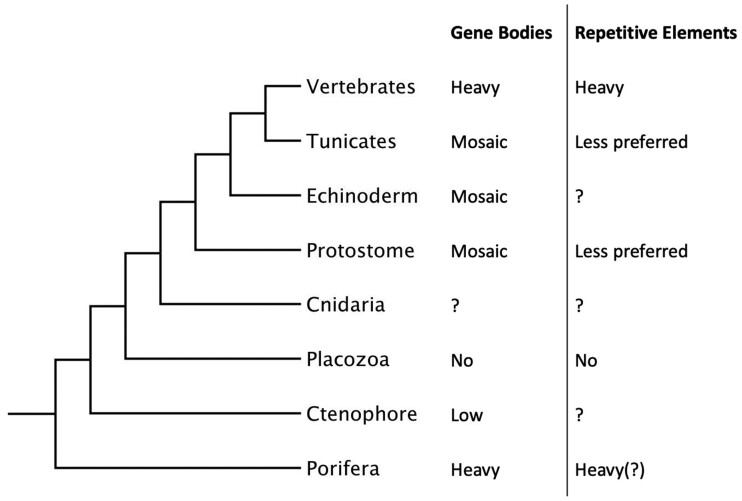
Methylation on the gene bodies and repetitive elements vary greatly across the tree of animals (Gavery and Roberts 2010; Hunt et al. 2010; Zemach et al. 2010; Glastad et al. 2011; Nanty et al. 2011; Sarda et al. 2012; Falckenhayn et al. 2013; Dabe et al. 2015).

CpG methylation on the gene bodies (transcription units) and repetitive elements vary greatly across Metazoa. In most bilaterian invertebrates examined, methylation is more concentrated at gene bodies than transposable elements (TEs) (Suzuki et al. 2007; Feng et al. 2010; Zemach et al. 2010) (fig. 1). Vertebrates, on the other hand, consistently methylate their genomes globally regardless of TE content (Zemach et al. 2010). Although defense against genome parasites has been proposed as the ancestral function of DNA methylation in eukaryotes (Bestor 1990), the varied patterns observed in vertebrates and bilaterian invertebrate suggest a complicated evolutionary history in Metazoa, and Sarda et al. (2012) among others argued that gene body methylation (GbM) was the ancestral DNA methylation pattern in animals. A recent study on sponge methylation showed heavy methylation on TEs as well as gene bodies, comparable to vertebrates, suggesting either convergence or multiple loss of this global methylation pattern (de Mendoza et al. 2019).

In bilaterian invertebrates, methylated and unmethylated gene bodies are typically found at approximately similar frequency, a pattern usually described as “bimodal.” Notably, genes with different modes of methylation status have been found to differ in functional enrichment, expression levels, and plasticity. Genes with high GbM tend to serve housekeeping functions with high and stable expression. Those with low GbM tend to function in stress response and developmental regulation, and exhibit relatively low, more plastic and more tissue or condition specific expression (Suzuki et al. 2007; Zemach et al. 2010; Glastad et al. 2014; Dimond and Roberts 2016).

GbM is functionally important as exemplified by studies in the honey bee (Kucharski et al. 2008), the Pacific oyster (Riviere et al. 2013), and the moon jellyfish (Fuchs et al. 2014). GbM is thought to repress spurious transcription initiation, which could allow for more efficient transcription elongation. This inference is consistent with the correlation between GbM and expression level and plasticity noted above. More direct evidence comes from a study in the anemone *Aiptasia* where genes with heavy GbM showed less intragenic promoter activity (Li et al. 2018). Aside from repressing intragenic promoters, Flores et al. (2012) proposed that GbM regulates alternative splicing. All these observations suggest the functional importance of GbM in certain circumstances, but confirmation of specific functions, are few and the generality of these functions across taxa is not well understood.

Interestingly, a number of Metazoan species, including the nematode *Caenorhabditis elegans*, and the fruit fly *Drosophila melanogaster* lack cytosine methylation (Wenzel et al. 2011; Takayama et al. 2014). Loss of cytosine methylation potentially relates to particular developmental modes, such as mosaic development where cell fates are determined early on, or shortened life cycles associated with transient larval resources (Bestor 1990; Canestro et al. 2007). Such methylation loss often correlates with drastic genome compaction such as occurs in parasitic animals. Understanding the context and consequences of these losses is important for understanding the evolution of global methylation patterns.

DNMTs, a shared attribute of crown group eukaryotes, mediate methylation of CpG sites, among which DNMT1and 3 are specific to Metazoa (Zemach et al. 2010). DNMT1 maintains DNA methylation and restores symmetric methylation of hemimethylated CpGs after DNA duplication, whereas DNMT3 methylates previously unmethylated CpG sites (Holz-Schietinger et al. 2011). DNMT1 and DNMT3 both have undergone frequent duplications and losses across Metazoa, and the loss of DNMTs typically concur with loss of methylation (Tweedie et al. 1997; Field et al. 2004; Goll et al. 2006; Yi and Goodisman 2009; Wenzel et al. 2011).

Although DNMTs are the writers of DNA methylation, this information is read by MBDs. Invertebrate genomes commonly possess only one gene in the MBD family, MBD2/3, whereas the gene family has expanded in mammals (Hendrich and Tweedie 2003; Cramer et al. 2017). This expansion is evolutionarily coincident with the increase of global methylation documented more broadly in the vertebrates (Hendrich and Tweedie 2003).

Methylated cytosines spontaneously deaminate to uracils more readily than unmethylated cytosines (Duncan and Miller 1980). Due to this hypermutability, sequences that are historically highly methylated in the germline over evolutionary time become depleted in CpG. CpG depletion has been shown to correlate with direct measures of somatic DNA methylation using methods such as bisulfite sequencing, making it a reliable first order approximation of DNA methylation (Bock and Lengauer 2008; Elango and Yi 2008; Xiang et al. 2010; Park et al. 2011; Sarda et al. 2012; Keller et al. 2016). Thus, depletion of CpG in a genomic region, often calculated as the ratio of observed to expected CpG (CpG_o/e_) as discussed in Materials and Methods, provides a proxy metric for methylation in the absence of bisulfite sequencing data. Consequently, a number of studies have used CpG depletion to understand phylogenetic aspects of DNA methylation in animals, yet application of this approach outside of Bilateria is minimal (Yi and Goodisman 2009; Sarda et al. 2012; Dixon et al. 2014; Aliaga et al. 2019).

Cnidaria, the sister group to Bilateria, encompasses species with diverse morphology, life history, coloniality, and skeletonization; while feeding modes include parasitism, predation, and photosymbiosis. Therefore, not only are Cnidaria interesting in comparison to Bilateria, they also merit comparative investigation among themselves due to this diversity, antiquity, and as an ancient parallel evolutionary radiation to Bilateria. Yet, previous studies on Cnidarian DNA methylation have been limited to the class Anthozoa including *Nematostella vectensis, Aiptasia*, and several coral species, (Zemach et al. 2010; Dixon et al. 2014, 2016; Dimond and Roberts 2016; Li et al. 2018), and the parasitic clade Myxozoa (Kyger et al. 2020). We survey the extent and distribution of DNA methylation in Cnidaria, to better understand how the variation of methylation relates to their diverse biology, and to inform reconstruction of the state of DNA methylation in the last common ancestor of Eumetazoa.

## Results

**Fig. 2.**
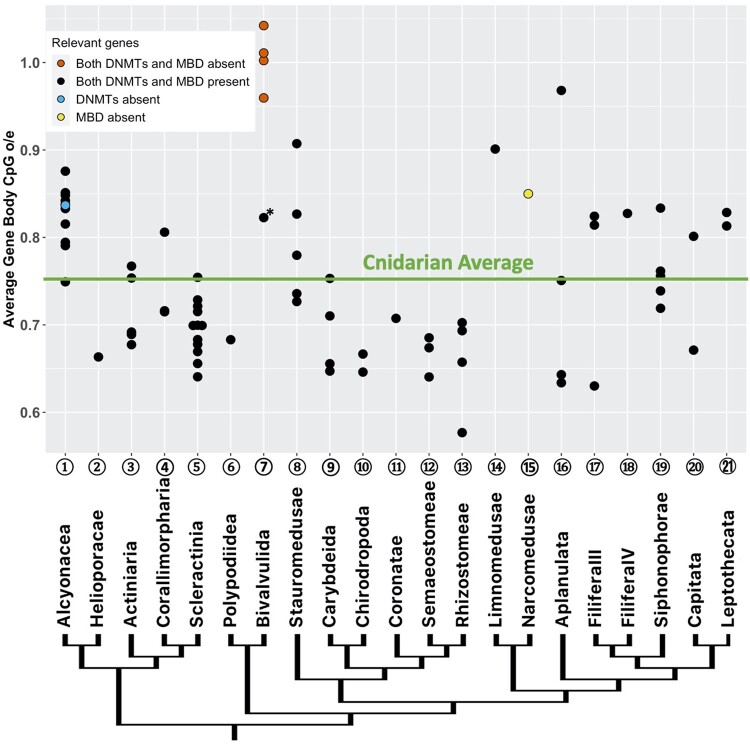
Average gene body CpG_o/e_ of each cnidarian species organized in ordinal rank groups. Green horizontal line indicates the average among all species. Phylogenetic relationship between the major cnidarian groups rendered from Kayal et al. (2018) and Bentlage and Collins (2021). Circled numbers correspond to figure 4 and table 1. The species marked by the asterisk is *Myxobolus pendula*, where the relative low average gene body CpG_o/e_ compared with the others in the class is possibly due to contamination from the fish host, which is discussed in detail in Results.

**Fig. 3.**
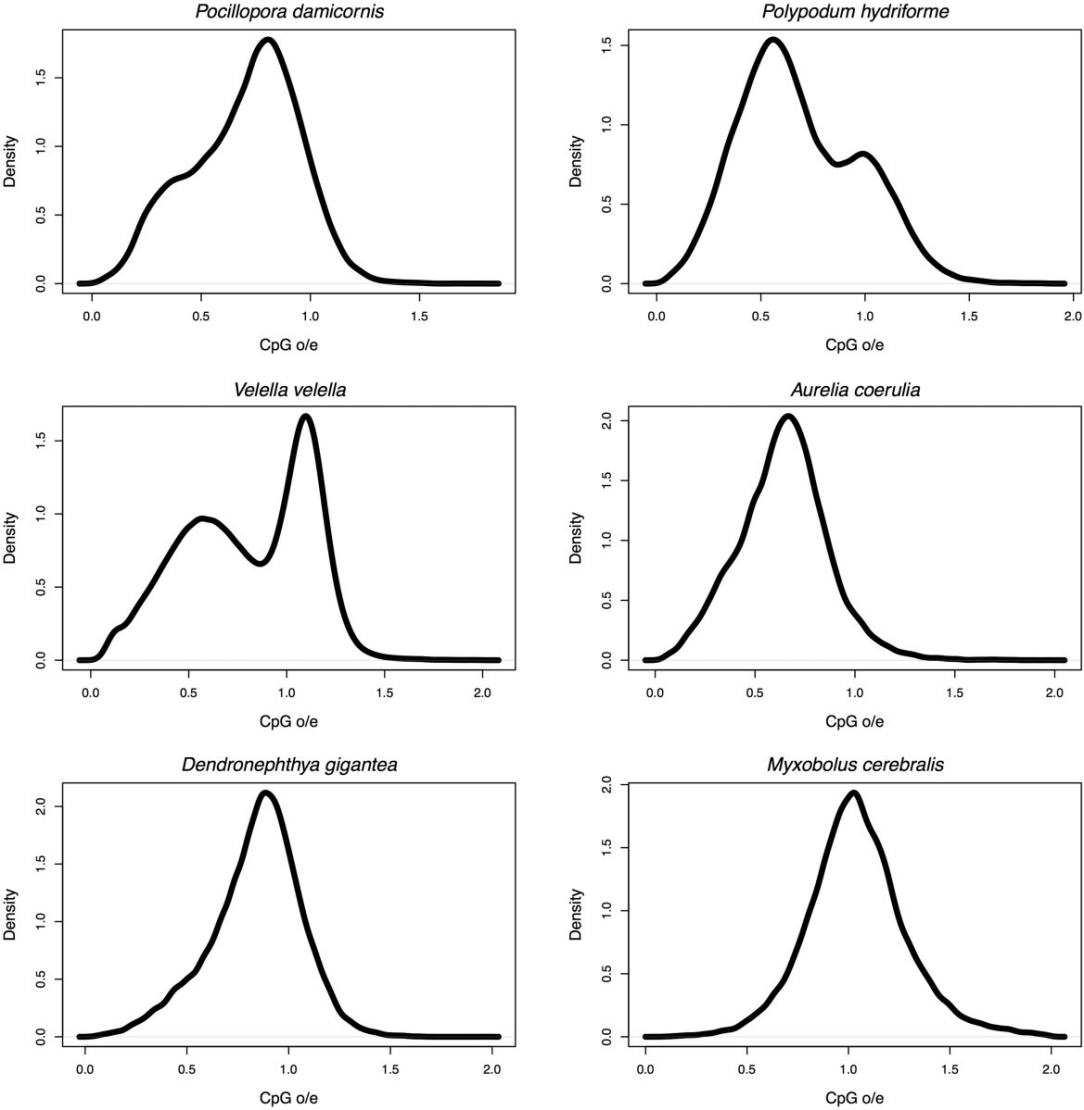
Density plots of gene body CpG_o/e_ of selected species as examples. The discrete bimodality typically found in bilaterian invertebrates occurs only in 11 of the 41 cnidarian species where CpG_o/e_ levels indicate substantial methylation. Among the six examples shown, *Polypodum and Velella* qualified as bimodal by Notos (and all taxa were classified as multimodal by mclust due to its sensitive nature). To further explore and group these patterns we conduct a clustering exercise (fig. 4), and a comparison of methylation in conserved and less conserved genes (table 2). *Pocillopora* and *Dendronephthya* are anthozoan corals; *Velella* is a hydrozoan, “by-the-wind sailor”; *Polypodum* belongs to the group Polypodiozoa, a sturgeon parasite, and *Myxobolus* is a member of Myxozoa which parasitizes bony fish; *Aurelia* is a scyphozoan jellyfish, “moon jelly.”

**Fig. 4.**
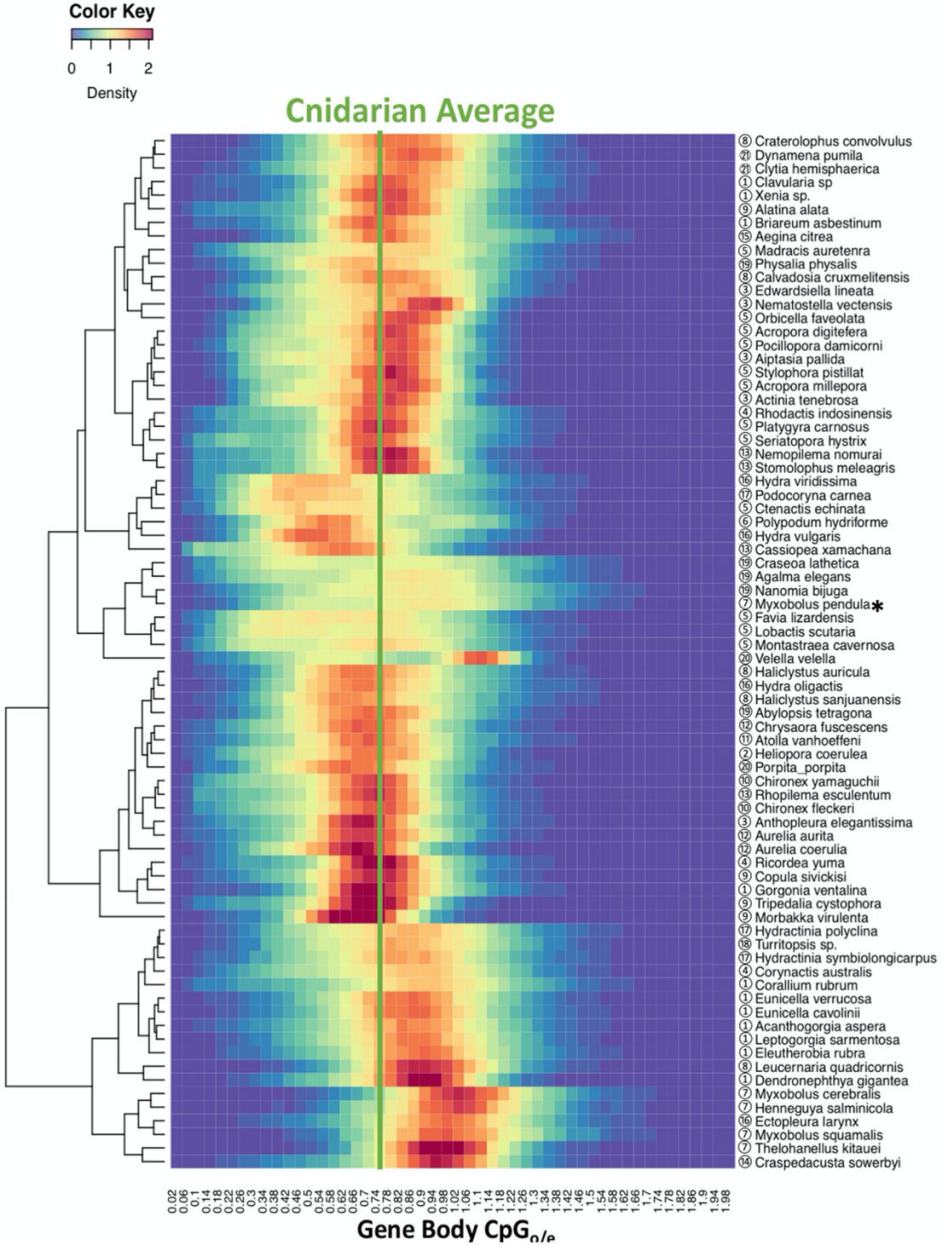
Heatmap of gene body CpG_o/e_ distribution patterns in Cnidaria. Circled numbers next to the species names correspond to figure 2 and table 1. The species marked by the asterisk is *Myxobolus pendula*, where the distribution pattern might have been affected by possible contamination from the fish host, which is discussed in detail in Results.

Forty-one of the 76 species examined in this study show an average gene body CpG_o/e_ lower than 0.75 (table 1) consistent with substantial methylation (Aliaga et al. 2019), suggesting prevalent GbM in Cnidaria. The average CpG_o/e_ of gene bodies varies greatly across the phylum, spanning from 0.58 in the jellyfish *Cassiopea xamachana* to 1.04 in the parasite *Myxobolus cerebralis* (table 1 and fig. 2). Only 11 of the 41 species with substantial gene body CpG depletion showed discrete bimodal distribution patterns of CpG_o/e_ based on analysis by Notos, a kernel density estimation (KDE) method, in contrast to most bilaterian invertebrates (fig. 3 and supplementary fig. S1, Supplementary Material online; Bulla et al. 2018). Additionally, we assessed the distribution patterns using Gaussian mixture models, which classified most species as multimodal due to the complex nature of the data. We will address these differences in results further in Discussion. Clustering of frequencies of CpG_o/e_ distribution across all gene bodies revealed differing patterns of methylation (fig. 4). Many, but not all, clades show similar patterns, including Alcyonacea (soft corals), Scleractinia (hard corals), Myxozoa (parasites), and Scyphozoa (true jellies) and Cubozoa (box jellies).

**Table 1:** Average Gene Body CpG_o/e_ and Repeat CpG_o/e_ of Species in This Study

Class	Ordinal Group	Species	Mean Gene Body CpG_o/e_	Mean Repeat CpG_o/e_
Anthozoa	Actiniaria 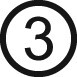	*Actinia tenebrosa*	0.69	0.90
		*Aiptasia pallida*	0.69	0.98
		*Anthopleura elegantissima*	0.68	
		*Edwardsiella lineata*	0.758	
		*Nematostella vectensis*	0.77	
	Alcyonacea 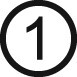	*Acanthogorgia aspera*	0.83	
		*Briareum asbestinum*	0.79	
		*Clavularia* sp.	0.82	
		*Corallium rubrum*	0.84	
		*Dendronephthya gigantea*	0.84	1.03
		*Eleutherobia rubra*	0.88	
		*Eunicella cavolinii*	0.85	
		*Eunicella verrucosa* ^a^	0.84	
		*Gorgonia ventalina*	0.75	
		*Leptogorgia sarmentosa*	0.85	
		*Xenia* sp.	0.79	
	Corallimorpharia 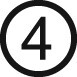	*Corynactis australis*	0.81	
		*Rhodactis indosinensis*	0.71	
		*Ricordea yuma*	0.72	
	Helioporacea 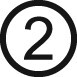	*Heliopora coerulea*	0.66	
	Scleractinia 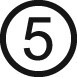	*Acropora digitefera*	0.70	0.90
		*Acropora millepora*	0.72	0.91
		*Ctenactis echinata*	0.64	
		*Favia lizardensis*	0.66	
		*Lobactis scutaria*	0.67	
		*Madracis auretenra*	0.75	
		*Montastraea cavernosa*	0.68	
		*Orbicella faveolata*	0.73	0.93
		*Platygyra carnosus*	0.72	
		*Pocillopora damicornis*	0.70	0.87
		*Seriatopora hystrix*	0.68	
		*Stylophora pistillata*	0.70	0.92
Hydrozoa	Aplanulata 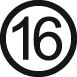	*Ectopleura larynx*	0.97	
		*Hydra oligactis*	0.75	
		*Hydra viridissima*	0.64	
		*Hydra vulgaris*	0.63	1.03
	Capitata 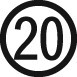	*Porpita porpita*	0.67	
		*Velella velella*	0.80	
	Filifera III 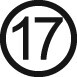	*Hydractinia polyclina*	0.82	
		*Hydractinia symbiolongicarpus*	0.81	
		*Podocoryna carnea*	0.63	
	Filifera IV 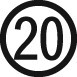	*Turritopsis* sp.	0.83	
	Leptothecata 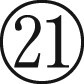	Clytia hemisphaerica	0.81	
		Dynamena pumila	0.83	
	Limnomedusae 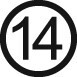	*Craspedacusta sowerbyi*	0.90	
	Narcomedusae 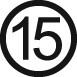	*Aegina citrea* ^b^	0.85	
	Siphonophorae 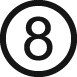	*Abylopsis tetragona*	0.72	
		*Agalma elegans*	0.76	
		*Craseoa lathetica*	0.74	
		*Nanomia bijuga*	0.83	
		*Physalia physalis*	0.76	
Cubozoa	Carybdeida 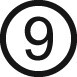	*Alatina alata*	0.75	
		*Copula sivickisi*	0.71	
		*Morbakka virulenta*	0.65	0.66
		*Tripedalia cystophora*	0.66	
	Chirodropoda 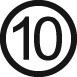	*Chironex fleckeri*	0.67	
		*Chironex yamaguchii*	0.65	
Scyphozoa	Coronatae 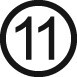	*Atolla vanhoeffeni*	0.71	
	Rhizostomeae 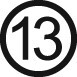	*Cassiopea xamachana*	0.58	0.65
		*Nemopilema nomurai*	0.69	
		*Rhopilema esculentum*	0.66	
		*Stomolophus meleagris*	0.70	
	Semaeostomeae 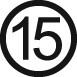	*Aurelia aurita*	0.69	
		*Aurelia coerulia*	0.649	0.74
		*Chrysaora fuscescens*	0.67	
Staurozoa	Stauromedusae 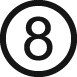	*Calvadosia cruxmelitensis*	0.78	
		*Craterolophus convolvulus*	0.83	
		*Haliclystus auricula*	0.74	
		*Haliclystus sanjuanensis*	0.73	
		*Lucernaria quadricornis*	0.91	
Myxozoa	Bivalvulida 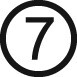	*Henneguya salminicola* ^c^	1.01	
		*Myxobolus cerebralis* ^c^	1.04	
		*Myxobolus pendula* ^c^	0.82	
		*Myxobolus squamalis* ^c^	1.00	0.62
		*Thelohanellus kitauei* ^c^	0.96	1.07
Polypodiozoa	Polypodiidea 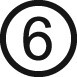	*Polypodum hydriforme*	0.68	

Note.—Circled numbers correspond to figures 2 and 4.

a Species in which DNMTs are absent.

b Species in which MBDs are absent.

c Species in which both DNMTs and MBDs are absent.

DNMT1, DNMT3, and MBDs were found in most species examined, with six exceptions. None of the DNMTs and MBDs was recovered in the myxozoan parasites *Myxobolus cerebralis, Myxobolus squamalis, Henneguya salminicola, and Thelohanellus kitauei*. DNMTs were also not found in the anthozoan *Eunicella verrucosa*, and MBDs were not found in the hydrozoan *Aegina citrea* (table 1 and fig. 2). All four myxozoan species mentioned above show little to no sign of CpG depletion on the gene bodies, indicating a lack of methylation consistent with absence of the enzymes. DNMTs were initially found in *Myxobolus pendula*, but our phylogenetic analysis suggests they are more closely related to DNMTs found in the zebrafish than the other Cnidarians sampled here, suggesting the transcripts most likely originated from the fish host (supplementary fig. S2, Supplementary Material online). This possibility of contamination could also explain the different pattern of gene body CpG_o/e_ distribution in *Myxobolus pendula* than the other myxozoans (figs. 2 and 4). *Eunicella verrucosa* and *Aegina citrea* also show elevated levels of gene body CpG_o/e_. However, several additional species also have high gene body CpG_o/e_ despite the presence in their genomes of DNMTs and MBDs. For instance, the hydrozoan *Ectopleura larynx* provides an example of implicit limited methylation despite the presence of the requisite genes for methylation (table 1 and fig. 2).

To compare CpG_o/e_ of conserved genes to nonconserved genes, ten representative species with high-quality transcriptomes were selected covering the major cnidarian groups. In nine of the ten species, orthologous genes shared by the ten selected species were found to have significantly lower CpG_o/e_ than nonorthologous genes, with the exception of *Calvadosia* (table 2). In eight of the ten species, orthologous genes are enriched in those with lower CpG_o/e_ (supplementary table S2, Supplementary Material online) with *Calvadosia and Clytia* being the two exceptions, both of which have overall average gene body CpG_o/e_ above 0.75 suggesting minimal methylation (table 1). These observations indicate that more conserved genes have higher methylation, consistent with previous findings in other taxa (Sarda et al. 2012). Such comparison of conserved genes and nonconserved genes is likely less biologically meaningful in species with low methylation globally such as *Calvadosia* and *Clytia*. For each species pair, genes that are low in CpG_o/e_ (high in methylation) in both species are overrepresented in pairwise orthologs (supplementary table S3, Supplementary Material online). Additionally, differentially expressed genes in *Aurelia coerulia* reported by Gold et al. (2019) have significantly higher CpG_o/e_ than nondifferentially expressed genes (*P* < 2.2e-16; Welch two sample *t*-test).

**Table 2:** CpG_o/e_ of Ten-Way Orthologous Genes versus Nonorthologous Genes in Each Species

Species	Mean CpG_o/e_ of Nonorthologous Genes	Mean CpG_o/e_ of Ten-Way Orthologous Genes	*t* stat.	*P* value
*Acropora digitifera*	0.7093909	0.6666281	15.794	2.2e-16
*Aiptasia pallida*	0.7152435	0.6313672	27.393	2.2e-16
*Alatina alata*	0.7597698	0.7381270	11.037	2.2e-16
*Aurelia coerulia*	0.6475442	0.6267479	6.8606	7.135e-12
*Calvadosia cruxmelitensis*	0.7769339	0.8065653	−15.144	2.2e-16
*Clytia hemisphaerica*	0.8164449	0.8091184	3.2878	0.001012
*Hydra vulgaris*	0.6437276	0.6156787	8.1367	4.315e-16
*Morbakka virulenta*	0.6508604	0.6392557	5.6923	1.271e-08
*Nematostella vectensis*	0.7860412	0.7203910	25.841	2.2e-16
*Physalia physalis*	0.7628115	0.6970781	16.648	2.2e-16

Note.—With the exception of *Calvadosia*, orthologous genes have higher methylation compared with non-ten-way orthologous genes in each species.

**Fig. 5.**
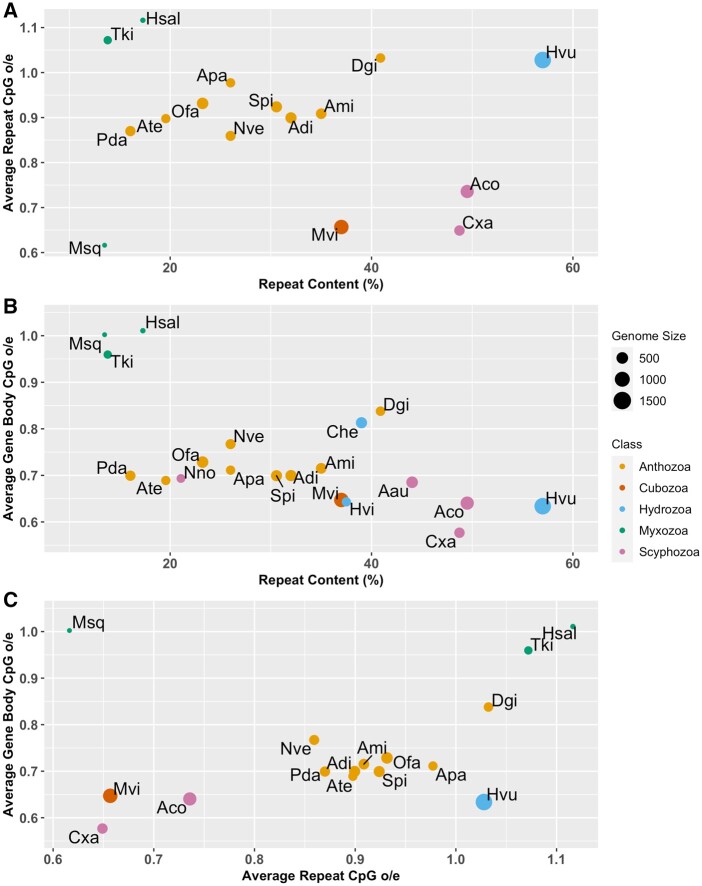
Both repeat and gene body CpG_o/e_ correlate with repeat content. (*A*) The average CpG_o/e_ of repeats is negatively correlated with the repeat content of the genome, with two notable exceptions *(Hydra vulgaris* and *Myxobolussquamalis*). (*B*) The average gene body CpG_o/e_ is negatively correlated with repeat content. (*C*) Gene body CpG_o/e_ and repeat CpG_o/e_ are positively correalted with each other while gene body CpG_o/e_ are generally lower than repeat CpG_o/e_, indicating gene bodies are preferred targets of methylation. Genome sizes in Mb. Aau, *Aurelia aurita*; Aco, *Aurelia coerulia*; Adi, *Acropora digitefera*; Ami, *Acropora millepora*; Apa, *Aiptasia pallida*; Ate, *Actinia tenebrosa*; Che, *Clytia hemisphera*; Cxa, *Cassiopea xamachana*; Dgi, *Dendronephthya gigantea*; Hsal, *Henneguya salminicola*; Hvu, *Hydra vulgaris*; Msq, *Myxobolus squamalis*; Mvi, *Morbakka virulenta*; Nve, *Nematostella vectensis*; Ofa, *Orbicella faveolata*; Pda, *Pocillopora damicornis*; Spi, *Stylophora pistillata*; Tki, *Thelohanellus kitauei*.

CpG_o/e_ of the repeats proved higher than that of gene bodies in 14 out of the 15 species surveyed for both, indicating that repeats are generally less preferred sites of methylation in Cnidaria; an intracellular parasite *Myxobolus squamalis* was the exception (fig. 5). Both gene body and repeat CpG_o/e_ negatively correlate with repeat content (fig. 5*A* and *B*) (Gene body: *t*=−3.4576, *P*=0.003008; Repeat: *t*=−3.6855, *P*=0.003591; Pearson correlation) and positively correlate with each other (fig. 5*C*) (*t* = 5.0099, *P*=0.0003963, Pearson correlation). The outliers *Hydra vulgaris* and *Myxobolus squamalis* were excluded from the statistical tests, as will be addressed in Discussion.

## Discussion

### The LCA of Eumetazoa Likely Preferentially Methylated Gene Bodies Relative to Repetitive Elements

Sarda et al. (2012) argued that gene body methylation is the ancestral methylation mode in Metazoa. Consistent with previous studies on *Nematostella* and *Acropora* (Zemach et al. 2010; Dixon et al. 2014), our results suggest pervasive higher GbM than repeat methylation across cnidaria. This study, combined with similar data for most nonvertebrate Bilateria, further supports GbM as the ancestral condition in Cnidaria, Bilateria, and the Eumetazoa.

### Patterns of GbM in Cnidaria: Lack of Discrete Bimodality

Most species in our study did not exhibit the classic bimodal distribution of GbM found in bilaterian invertebrates where the two classes of genes significantly differ in terms of methylation status, functional enrichment, and expression plasticity (Zemach et al. 2010; Glastad et al. 2014; Dimond and Roberts 2016). However, our results show that conserved genes which are presumably expressed constitutively have higher methylation (table 2). Thus, cnidarian GbM appears to play a similar role in regulation of gene expression to that observed in bilaterian invertebrates, with the caveat that this pattern appears less pronounced or discretely bimodal in Cnidaria.

In addition to Notos, a KDE-based method, we further assessed the GbM distribution patterns using Gaussian mixture models, and models with three or more components best fit the GbM data for the vast majority of the species in question (fig. 3 and supplementary fig. S1, Supplementary Material online) (Fraley and Raftery 2003). Gaussian mixture models have previously identified three or more components in *Acropora* and some bilaterian animals as well (Dixon et al. 2016). The different results from Notos and Gaussian mixture models highlight the complexity of the data, which is further explored via the clustering analysis (fig. 4). From the perspective of these data, the default criteria set by Notos appear too stringent. In contrast, Gaussian mixture models are less interpretable due to excessive sensitivity. We note that assessing the complex distribution patterns of GbM using parameters previously applied to another group, in this case Bilateria, provides an arbitrary and incomplete picture of the variation. Nevertheless, GbM is partially correlated with gene conservation in Cnidaria in a broad suite of species including those that are not diagnosed as formally bimodal using Notos (table 2). Thus, we do find evidence of similar processes operating in Bilateria and Cnidaria relative to methylation and gene expression and function. However, our results also highlight substantial differences between Bilateria and Cnidaria. Thus, caution is needed when choosing methods to describe distribution patterns of gene body methylation across previously unexamined groups.

### Patterns of GbM in Cnidaria: Lack of CpG_o/e_ Higher Than 1

In addition to less-pronounced bimodality, most cnidarian species have very few genes with CpG_o/e_ higher than 1 (fig. 4 and supplementary fig. S1, Supplementary Material online). This contrasts with bilaterian invertebrates where modes well above 1 are often observed (e.g., Park et al. 2011). These values above 1 may suggest general compensatory mechanisms that restore CpGs on gene bodies in bilaterian invertebrates that do not exist in Cnidaria. The presence of compensatory mechanisms could also explain the stronger bimodality observed in invertebrate Bilateria. There are a number of DNA repair mechanisms that could play a role in this, however of particular interest are the DNA repair functions exhibited by some MBD proteins in conjunction with other molecules (Watanabe et al. 2003; Wu et al. 2003). Further work in this area could prove revealing regarding the broad evolution of mechanisms controlling GbM and its role in epigenesis.

### Patterns of GbM in Cnidaria: Complex Patterns Partially Reflecting Taxonomy and Life History

GbM varies dramatically across cnidarian taxa indicating a complex evolutionary history (figs. 2 and 4). This is not surprising given the antiquity of Cnidaria as a whole; they are sister to Bilateria with a Precambrian origin, and the separation of the major cnidarian groups occurred over 500 Ma (Khalturin et al. 2019). By sampling widely in Cnidaria, we show that the GbM pattern is quite complex (figs. 2 and 4) and is not consistent within phyla or classes. However, multiple orders within particular class level taxa show consistent GbM, which we discuss later. In contrast, in insects, a single class, there is relatively consistent GbM across ordinal rank taxa with a trend toward less methylation in the advanced (Holometabolous) insects (Provataris et al. 2018). However, it is important to note that the insects are a much younger group that radiated in the late Paleozoic, thus comparison between orders in the two groups may not be appropriate.

In our data, GbM shows some relationship to life history complexity. Cubozoa and Scyphozoa, both have complex life cycles that include a medusa phase, and both have higher levels of GbM relative to the other groups (Cubozoa and Scyphozoa are groups ⑨–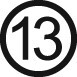; fig. 2). Previous work in Scyphozoan jellyfish *Aurelia aurita* showed that disrupting DNMT1 halts strobilation, the transition from the sessile polyp stage to free-swimming medusa stage, suggesting that cytosine methylation is functionally important in regulating life history transitions (Fuchs et al. 2014). On the other hand, Myxozoa which includes endoparasitic species with reduced complexity in life history (as summarized in Kent et al. [2001]) have high CpG_o/e_ suggesting the absence of GbM. In contrast, the sister group to Myxozoa, *Polypodum hydriforme* shows one of the lowest gene body CpG_o/e_ indicating high methylation. It is a parasitic species, but with an elaborate life history including a free-living stolon stage (Raikova 1994; Kayal et al. 2018).

On the other hand, the correlation between life history complexity and GbM is less clear in Hydrozoa, where dramatic differences occur even within families. The two taxa studied from the Capitata taxon are in the family Porpitidae, the by-the-wind sailors which have an unusual mode of life as colonies of medusae function as surface vessels. Despite the similarity in this mode of life and familial relationship, the species studied have very different GbM. Within the Aplanulata group (which includes the well-known freshwater genus *Hydra* and lacks a planula phase), the three *Hydra* species samples have high GbM, whereas the other Aplanulata genus sampled, *Ectopleura*, has low GbM. Similarly, within the Filifera III group (Bentlage and Collins 2021), both members of *Hydractinia* examined have low GbM, whereas that of *Podocoryne* is much higher.

In examining evolution of methylation implied by these patterns it is important to consider the antiquity and diversity of Hydrozoa. The modern species in the genus Hydra diverged 100–200 Ma in the Mesozoic prior to the K/T extinction (Schwentner and Bosch 2015; Wong et al. 2019), whereas a derived hydrozoan fossil from 520 Myr in the Cambrian suggests that the hydrozoan crown group is at least that old (Muscente et al. 2016; Song et al. 2021). In addition, Hydrozoa is the most diverse cnidarian group and higher order relationships within the hydrozoa are only now coming into focus (Bentlage and Collins 2021). Recent examination of the better fossilized Anthozoa suggests a still greater antiquity for the cnidarian radiation as a whole (McFadden et al. 2021). Our results suggest that DNA methylation has gone through dynamic changes in the evolutionary history of Cnidaria.

Others have noted that life history complexity and DNA methylation are not always correlated. For example, sponges have surprisingly high levels of GbM even though they are seemingly among the simplest forms of animals (de Mendoza et al. 2019). Thus, our results suggest that life history complexity appears associated with DNA methylation in cnidarians, but it does not appear to be the only factor. More detailed studies of GbM in Medusozoa would help illuminate the role of DNA methylation in the life history evolution across the group.

### Patterns of GbM in Cnidaria: Absence of GbM in Parasitic Species

We present several cases of absence of CpG methylation in Myxozoa (fig. 2), a clade comprised parasitic species. Kyger et al. (2020) showed the absence of cytosine methylation in *Henneguya salminicola* and one additional Myxozoan species using bisulfite sequencing, and our result suggests that both DNMT1 and DNMT3 are absent in the entire clade. Aside from the reduction of life history complexity, this might also be due to the massively reduced genome sizes of myxozoans (Chang et al. 2015). Lechner et al. (2013) presented a positive correlation between genome size and DNA methylation across a range of metazoan species. However, in other contexts small genome size does not automatically lead to loss of methylation. *Plasmodium falciparum* maintains methylation, albeit in a different nucleotide context, in a much smaller genome than myxozoans (Ponts et al. 2013). Therefore, care must be taken in strong inferences of complete loss of methylation in small myxozoan genomes. Moreover, since Myxozoa is a diverse group of parasites inhabiting many teleost lineages, wider sampling might yield further insights.

Our results also support low GbM across the species of Alcyonacea analyzed. Consistent with low methylation, DNMTs were not recovered in the transcriptome of one of the species (*Eunicella verrucosa)*, suggesting either the expression of the enzymes are extremely low, or they have been lost in the genome (fig. 2).

### Methylation on Repetitive Elements Correlates with Repeat Content

Previously Zemach et al. (2010) showed that *Nematostella* had less methylation on repeats than gene bodies using bisulfite sequencing. Our results add to this and are consistent with studies in other invertebrate phyla (Feng et al. 2010; Xiang et al. 2010; de Mendoza et al. 2019).

The correlation between overall methylation levels and repeat content has been unclear, although it is well documented that metazoan genome size and overall methylation level are positively correlated (Lechner et al. 2013; Zhou et al. 2020). For example, the pufferfish *Tetraodon nigroviridis* has very low repeat content, yet it still exhibits the typical vertebrate hypermethylation pattern (Zemach et al. 2010); the silk moth *Bombyx mori* has a transposon-rich genome, yet shows very low level of overall DNA methylation, as well as low GbM (Xiang et al. 2010). In the sponge *Amphimedon queenslandica* the genome is hypermethylated as in vertebrates, with interspersed repeats making up 35% of the genome. These repeats are heavily methylated approaching the vertebrate condition (de Mendoza et al. 2019). In this study, we found significant correlation between repeat methylation and gene body methylation, and both are positively correlated with repeat content (fig. 5). Our results suggest that global methylation, GbM, and repeat methylation are correlated with repeat content in Cnidaria, however, there are two exceptions worth discussing.

The *Hydra vulgaris* genome is drastically bigger than typical cnidarian genomes as the result of the invasion and expansion of a single class of TEs (Wong et al. 2019), and it has one the lowest gene body CpG_o/e_ (fig. 2), indicating heavy methylation on the gene bodies. Yet the repeats showed no signs of CpG methylation (fig. 5). This may relate to the young age and rapid expansion of repeats as young repeats tend to be less methylated (Lechner et al. 2013). Alternatively, conversion of methylated C to T in recently methylated repeats may not have caught up with current methylation yielding a minimal departure from expected CpG frequency. Both of these could be tested by experimentally measuring DNA methylation on the repeats by methods such as bisulfite sequencing. Lastly, *Hydra* could have activated other mechanisms to defend against the TEs that do not involve CpG methylation (de Mendoza et al. 2019).

We observe an additional exception to the positive correlation between repeat content and methylation on the various genomic elements in *Myxobolus squamalis*, a myxozoan parasite which had no signs of methylation on the gene bodies but significant methylation on the repeats (fig. 5). Preferential methylation of repeats over gene bodies is opposite the results from a wide range of invertebrates (Zemach and Zilberman 2010; Sarda et al. 2012) as well as the cnidarians reported here. It is even more surprising as no DNMT or MBD was found in the transcriptome or the genome of *Myxobolus squamalis* (table 1). It would also be interesting to conduct a bisulfite sequencing study on *Myxobolus squamalis* to verify that there is indeed cytosine methylation on the repeats. CpG_o/e_ has been shown to correlate well with experimentally determined methylation, thus *Myxobolus squamalis* provides quite an interesting system to follow up on regarding both CpG_o/e_ as a reliable proxy for CpG methylation given the methylation of repeats where DNMTs are not currently observed.

To our knowledge, the relationship between repeat content and DNA methylation on various elements including TEs and gene bodies has not been previously studied with the consistent sampling across a large group comparable to our study. Nevertheless, previous work with fewer taxa convey important related information; bisulfite sequencing in several fungal species revealed a positive correlation between repeat content, genome-wide methylation, and TE methylation similar to our results (Hosseini et al. 2020). Comparative studies investigating such relationships require well-annotated genomes in closely related species. Several cnidarian genera such as *Acropora* in Anthozoa and *Aurelia* in Scyphozoa (Dawson and Jacobs 2001) appear poised for exploration in this fashion. Future studies could take advantage of these resources to examine evolution of methylation on TEs and GbM in more closely related cnidarian taxon sets.

From this comprehensive study, we show that DNA methylation patterns have some lineage specificity, but are often subject to significant evolutionary change within current taxon sampling. We report for the first time that genes with CpG_o/e_ above 1 are rare in most cnidarians. This suggests the possibility of unknown compensatory mechanisms that restore CpG sites in bilaterian invertebrates that are absent in Cnidaria. In addition, the distribution of GbM across genes is less discretely bimodal in cnidarian taxa than it is in bilaterian invertebrates; nevertheless, we observe differences in GbM between conserved and differentially expressed genes. Thus, cnidarian GbM appears to also be involved in regulating gene expression. This builds on previous observations in two cnidarian taxa, *Nematostella* and *Acropora*, where GbM differs between stable and dynamically expressed genes (Dixon et al. 2014; Dimond and Roberts 2016). Intermediate levels of GbM appear likely to have been present in the last common ancestor of Eumetazoa. However, the incomplete agreement on the placement of Ctenophore and Placozoa in the Metazoa, and the limited methylation in these taxa makes it more difficult to be certain that the dramatically high methylation in sponges and vertebrates is a result of convergent evolution (Albalat 2008; Lechner et al. 2013; Dabe et al. 2015). Changes in GbM occur within several cnidarian orders, with parasitic taxa exhibiting dramatic methylation loss. We also note that scyphozoan and cubozoan jellyfish exhibit increased methylation and increased life history complexity, and increased repeat content is also associated with increased DNA methylation. However, neither life history changes nor evolution of repeat content appear sufficient to explain the patterns across the species studied. This work reveals a dynamic evolutionary history of DNA methylation among cnidarian groups and even within closely related clades. Our effort also points to several examples where bisulfite sequencing of a limited number of taxa would be revealing.

## Materials and Methods

### Measurement of CpG Depletion

CpG depletion is measured as the ratio of observed to expected CpG. This was calculated as *P*_CpG_/(*P*_C_×*P*_G_), where *P*_CpG_, *P*_C_, and *P*_G_ are the frequencies of CpGs, cytosines, and guanines respectively (Elango et al. 2009). Loss of CpG due to DNA methylation is associated with conversion to TpG (Duncan and Miller 1980). Thus, to confirm that CpG depletion is a result of methylation, we also measured the enrichment of TpG, where TpG_o/e_ was calculated in the same way.

### Data Sources

In total, this study analyzed data from 76 species spanning across seven classes and 21 ordinal groups. Data sources used are summarized in supplementary table S1, Supplementary Material online. All transcriptomes had BUSCO scores higher than 50% For taxa with both an available annotated genome and transcriptome, the transcriptome is used for gene body CpG_o/e_ as it was previously shown that measurement of CpG depletion using coding sequences and cDNAs better reflect experimentally determined methylation levels (Aliaga et al. 2019). Predicated gene models and transcripts that are shorter than 300 bp were excluded from subsequent analyses.

About ten species that have both transcriptomes and annotated genomes were selected for analyses of repetitive elements. Repetitive elements were identified de novo with RepeatScout and RepeatMasker (Smit and Hubley 2015; Smit et al. 2015). Repetitive elements longer than 50 bp and occur more than ten times in the genome were used for subsequent analyses. Repeats that overlap with predicted gene bodies were excluded. For all of these ten species, gene body CpG analysis was done using the transcriptomes, effectively excluding repeats that might reside in introns.

### Statistical Analyses of Gene Body CpG_o/e_ Distribution Modality

To estimate the number of components in the density distribution of gene body CpG_o/e_ in each species, we employed 1) the *Notos* tool, a KDE-based approach (Bulla et al. 2018), and 2) a model-based clustering approach using the mclust package (Fraley and Raftery 2003) followed by Bayesian information criteria to measure the fit of each model. Both were conducted in R (www.r-project.org).

### Search for DNMTs and MBDs

The presence of DNMT1, DNMT3, and MBDs in 76 species were screened via TBlastN searches and verified through reciprocal BLAST against the NCBI nonredundant nucleotide database. The queries used for DNMT1 are XP_012557244.1 (*Hydra vulgaris*) and XP_020612302.1 (*Orbicella faveolata*); the queries used for DNMT3 are XP_012561137.1 (*Hydra vulgaris*) and XP_015756999.1 (*Acropora digitifera*); the queries used for MBDs are XP_020895950.1 (*Aiptasia pallida*) and XP_020604760.1 (*Orbicella faveolata*).

To determine whether the DNMTs found in the parasite *Myxobolus pendula* are indeed from the cnidarian or from the fish host, we conducted phylogenetic analyses. DNMT1 and DNMT3 protein sequences were obtained using Transdecoder (https://github.com/TransDecoder/TransDecoder/wiki, last accessed February 4, 2022). The sequences were aligned using MUSCLE (Edgar 2004) and then manually curated. Phylogenetic trees were reconstructed using RAxML v8.2.12 (Stamatakis 2014).

### Ortholog CpG_o/e_ Analyses in Selected Species

Orthologs between *Acropora digitifera* (Anthozoa), *Aiptasia pallida* (Anthozoa), *Alatina alata* (Cubozoa), *Aurelia coerulia* (Scyphozoa), *Calvadosia cruxmelitensis* (Staurozoa), *Clytia hemisphaerica* (Hydrozoa), *Hydra vulgaris* (Hydrozoa), *Morbakka virulenta* (Cubozoa), *Nematostella vectensis* (Anthozoa), and *Physalia physalis* (Hydrozoa) were determined using Orthofinder version 2.4.0 (Emms and Kelly 2015, 2019) with Diamond protein alignment (Buchfink et al. 2015). These species were selected for their high-quality transcriptomes to yield a sufficient number of orthologs for our analyses. For *Alatina*, *Calvadosia*, *Clytia*, and *Physalia*, transcripts were first translated into protein sequences using Transdecoder version 5.3.0 with default settings (https://github.com/TransDecoder/TransDecoder/wiki, last accessed February 4, 2022).

For each species, CpG_o/e_ of ten-way orthologs was compared with genes that are not shared by all ten species. For each pair of species, CpG_o/e_ of single orthologs in each species is compared. 

## Supplementary Material

Supplementary data are available at *Genome Biology and Evolution* online.

## Supplementary Material

evab284_Supplementary_Data
