# Supplementary material for: A Broad Survey of Gene Body and Repeat Methylation in Cnidaria Reveals a Complex Evolutionary History
Source: Genome Biol Evol. 2022 Feb 1;14(2):evab284. doi: 10.1093/gbe/evab284 (PMC8857923; doi:10.1093/gbe/evab284)
Supplement: evab284_Supplementary_Data [file evab284_supplementary_data.zip › Supplemental_Materials_revision_211025.docx]

### **Supplemental Materials**

Table S1. Genomic/transcriptomic resources used in this study. Gray shaded species indicate predicted gene models used for gene body CpG_o/e_ analyses instead of transcripts.

| Class | Order | Species | Source for GbM analyses | Source for repeat analyses |
| --- | --- | --- | --- | --- |
| Anthozoa | Actiniaria | *Actinia tenebrosa* | GCF_009602425.1 | GCF_009602425.1 |
|  |  | *Aiptasia pallida* | Zapata *et al.* 2015 | GCF_001417965.1 |
|  |  | *Anthopleura elegantissima* | GBXJ00000000; GBYC00000000 |  |
|  |  | *Edwardsiella lineata* | Stefanik *et al.* 2014 |  |
|  |  | *Nematostella vectensis* | GCA_000209225.1 | GCA_000209225.1 |
|  | Alcyonacea | *Acanthogorgia aspera* | GETB00000000 |  |
|  |  | *Briareum asbestinum* | GHBD00000000 |  |
|  |  | *Clavularia sp.* | GHAW00000000 |  |
|  |  | *Corallium rubrum* | SRR1552944 |  |
|  |  | *Dendronephthya gigantea* | GCF_004324835.1 | GCF_004324835 |
|  |  | *Eleutherobia rubra* | GHFI00000000 |  |
|  |  | *Eunicella cavolinii* | SRR1324943 |  |
|  |  | *Eunicella verrucosa* | SRR1324944 |  |
|  |  | *Gorgonia ventalina* | SRR935083 |  |
|  |  | *Leptogorgia sarmentosa* | SRR1324968 |  |
|  |  | *Xenia sp.* | GHBC00000000 |  |
|  | Corallimorpharia | *Corynactis australis* | GELM00000000 |  |
|  |  | *Rhodactis indosinensis* | GELO00000000 |  |
|  |  | *Ricordea yuma* | GELN00000000 |  |
|  | Helioporacea | *Heliopora coerulea* | GFVH00000000 |  |
|  | Scleractinia | *Acropora digitefera* | GCF_000222465.1 | GCF_000222465.1 |
|  |  | *Acropora millepora* | GCF_004143615.1 | GCF_004143615.1 |
|  |  | *Ctenactis echinata* | Okubo *et al.* 2016 |  |
|  |  | *Favia lizardensis* | Okubo *et al.* 2016 |  |
|  |  | *Lobactis scutaria* | SRR2300562 |  |
|  |  | *Madracis auretenra* | Kayal *et al.* 2018 |  |
|  |  | *Montastraea cavernosa* | SRR2306543 |  |
|  |  | *Orbicella faveolata* | GCF_002042975.1 | GCF_002042975.1 |
|  |  | *Platygyra carnosus* | SRR402974-5 |  |
|  |  | *Pocillopora damicornis* | GCF_003704095.1 |  |
|  |  | *Seriatopora hystrix* | SRR2300678 |  |
|  |  | *Stylophora pistillata* | GCF_002571385.1 |  |
| Hydrozoa | Anthoathecata | *Ectopleura larynx* | Zapata *et al.* 2015 |  |
|  |  | *Hydra oligactis* | SRR040466-9 |  |
|  |  | *Hydra viridissima* | SRR040470-3 |  |
|  |  | *Hydra vulgaris* | GCF_000004095.1 | GCF_000004095.1 |
|  |  | *Hydractinia polyclina* | SRR923509 |  |
|  |  | *Hydractinia symbiolongicarpus* | Zapata et al. 2015 |  |
|  |  | *Podocoryna carnea* | Zapata et al. 2015 |  |
|  |  | *Porpita porpita* | GHBA00000000 |  |
|  |  | *Turritopsis sp* | Hasegawa *et al.* 2016 |  |
|  |  | *Velella velella* | GHAZ00000000 |  |
|  | Leptothecata | *Clytia hemisphaerica* | HAMU00000000 |  |
|  |  | *Dynamena pumila* | GHMC00000000 |  |
|  | Limnomedusae | *Craspedacusta sowerbyi* | SRR923472 |  |
|  | Narcomedusae | *Aegina citrea* | Zapata *et al.* 2015 |  |
|  | Siphonophorae | *Abylopsis tetragona* | Zapata *et al.* 2015 |  |
|  |  | *Agalma elegans* | Zapata *et al.* 2015 |  |
|  |  | *Craseoa lathetica* | Zapata *et al.* 2015 |  |
|  |  | *Nanomia bijuga* | Zapata *et al.* 2015 |  |
|  |  | *Physalia physalis* | Zapata *et al.* 2015 |  |
| Cubozoa | Carybdeida | *Alatina alata* | Zapata *et al.* 2015 |  |
|  |  | *Copula sivickisi* | GHBG00000000 |  |
|  |  | *Morbakka virulenta* | GHAF00000000 | GCA_003991215.1 |
|  |  | *Tripedalia cystophora* | GHAQ00000000 |  |
|  | Chirodropoda | *Chironex fleckeri* | SRR1819888 |  |
|  |  | *Chironex yamaguchii* | GHAX00000000 |  |
| Scyphozoa | Coronatae | *Atolla vanhoeffeni* | Zapata *et al.* 2015 |  |
|  | Rhizostomeae | *Cassiopea xamachana* | Kayal *et al.* 2018 | GCA_900291935.1 |
|  |  | *Nemopilema nomurai* | GHAR00000000 |  |
|  |  | *Rhopilema esculentum* | GEMS00000000 |  |
|  |  | *Stomolophus meleagris* | SRR1168418 |  |
|  | Semaeostomeae | *Aurelia aurita* | Brekhman *et al.* 2015 |  |
|  |  | *Aurelia coerulia* | Gold et al., 2019 | Gold et al., 2019 |
|  |  | *Chrysaora fuscescens* | SRR3180892 |  |
| Staurozoa | Stauromedusae | *Calvadosia cruxmelitensis* | Kayal *et al.* 2018 |  |
|  |  | *Craterolophus convolvulus* | Kayal *et al.* 2018 |  |
|  |  | *Haliclystus auricula* | HAHA00000000 |  |
|  |  | *Haliclystus sanjuanensis* | Kayal *et al.* 2018 |  |
|  |  | *Lucernaria quadricornis* | Kayal *et al.* 2018 |  |
| Myxozoa | Bivalvulida | *Henneguya salminicola* | GCA_009887335.1 |  |
|  |  | *Myxobolus cerebralis* | GBKL00000000 |  |
|  |  | *Myxobolus pendula* | SRR2472984;SRR2472987;SRR2472989 |  |
|  |  | *Myxobolus squamalis* | GHBR00000000 | GCA_010108815.1 |
|  |  | *Thelohanellus kitauei* | GCA_000827895.1 | GCA_000827895.1 |
| Polypodiozoa | Polypodiidea | *Polypodum hydriforme* | Kayal *et al.* 2018 |  |

Table S2. Genes that have orthologs in at least one other species are tested for enrichment in high or low methylation. A gene is categorized as ‘high’ methylation if the CpG_o/e_ is less than the median, and vice versa.

*Acropora digitifera*

|  | Low | High |
| --- | --- | --- |
| Orthologous | 12586 | 13471 |
| Non-orthologous | 7425 | 6541 |

X-squared = 3730.9, df = 3, p-value < 2.2e-16

*Aiptasia pallida*

|  | Low | High |
| --- | --- | --- |
| Orthologous | 11268 | 12281 |
| Non-orthologous | 3422 | 2409 |

X-squared = 10825, df = 3, p-value < 2.2e-16

*Alatina alata*

|  | Low | High |
| --- | --- | --- |
| Orthologous | 10616 | 13939 |
| Non-orthologous | 50872 | 47549 |

X-squared = 46824, df = 3, p-value < 2.2e-16

*Aurelia coerulia*

|  | Low | High |
| --- | --- | --- |
| Orthologous | 8277 | 9202 |
| Non-orthologous | 3250 | 2325 |

X-squared = 6295.1, df = 3, p-value < 2.2e-16

*Calvadosia cruxmelitensis*

|  | Low | High |
| --- | --- | --- |
| Orthologous | 14189 | 11822 |
| Non-orthologous | 36579 | 38946 |

X-squared = 24366, df = 3, p-value < 2.2e-16

*Clytia hemisphaerica*

|  | Low | High |
| --- | --- | --- |
| Orthologous | 16353 | 16024 |
| Non-orthologous | 36441 | 36770 |

X-squared = 15796, df = 3, p-value < 2.2e-16

*Hydra vulgaris*

|  | Low | High |
| --- | --- | --- |
| Orthologous | 6707 | 7070 |
| Non-orthologous | 4953 | 4590 |

X-squared = 15796, df = 3, p-value < 2.2e-16

*Morbakka virulenta*

|  | Low | High |
| --- | --- | --- |
| Orthologous | 10522 | 10791 |
| Non-orthologous | 3864 | 3595 |

X-squared = 6680.9, df = 3, p-value < 2.2e-16

*Nematostella vectensis*

|  | Low | High |
| --- | --- | --- |
| Orthologous | 13739 | 15012 |
| Non-orthologous | 6066 | 4792 |

X-squared = 8246.8, df = 3, p-value < 2.2e-16

*Physalia physalis*

|  | Low | High |
| --- | --- | --- |
| Orthologous | 10163 | 12618 |
| Non-orthologous | 20568 | 18113 |

X-squared = 4505.5, df = 3, p-value < 2.2e-16

Table S3. Single orthologs of various methylation status between pairs of species. In each species, the CpG_o/e_ of each gene is compared to the species median, and is categorized as ‘high’ if the CpG_o/e_ is less than the median, and vice versa. The rows are the methylation status in the first species, the columns are the methylation status in the second species.

*Aurelia coerulia* (Scyphozoa) *vs. Nematostella vectensis* (Athozoa)

*X-squared = 949.95, df = 3, p-value < 2.2e-16*

|  | Low | High |
| --- | --- | --- |
| Low | 275 | 534 |
| High | 146 | 1046 |

*Aurelia coerulia* (Scyphozoa) *vs. Hydra vulgaris* (Hydrozoa)

*X-squared = 90.261, df = 3, p-value < 2.2e-16*

|  | Low | High |
| --- | --- | --- |
| Low | 514 | 449 |
| High | 614 | 753 |

*Nematostella vectensis* (Anthozoa) *vs. Hydra vulgaris* (Hydrozoa)

*X-squared = 1140, df = 3, p-value < 2.2e-16*

|  | Low | High |
| --- | --- | --- |
| Low | 278 | 143 |
| High | 969 | 1119 |

*Acropora digitifera* (Anthozoa) *vs. Aurelia coerulia* (Scyphozoa)

*X-squared = 523.46, df = 3, p-value < 2.2e-16*

|  | Low | High |
| --- | --- | --- |
| Low | 343 | 526 |
| High | 300 | 968 |

*Acropora digitifera* (Anthozoa) *vs. Nematostella vectensis* (Anthozoa)

*X-squared = 1637, df = 3, p-value < 2.2e-16*

|  | Low | High |
| --- | --- | --- |
| Low | 647 | 260 |
| High | 500 | 1740 |

*Acropora digitifera* (Anthozoa) *vs. Hydra vulgaris* (Hydrozoa)

*X-squared = 595.96, df = 3, p-value < 2.2e-16*

|  | Low | High |
| --- | --- | --- |
| Low | 423 | 894 |
| High | 308 | 1069 |

*Alatina alata* (Cubozoa) *vs. Aurelia coerulia* (Scyphozoa)

*X-squared = 243.84, df = 3, p-value < 2.2e-16*

|  | Low | High |
| --- | --- | --- |
| Low | 563 | 437 |
| High | 503 | 934 |

*Alatina alata* (Cubozoa) *vs. Nematostella vectensis* (Anthozoa)

*X-squared = 1155.5, df = 3, p-value < 2.2e-16*

|  | Low | High |
| --- | --- | --- |
| Low | 267 | 629 |
| High | 220 | 1255 |

*Alatina alata* (Cubozoa) *vs. Hydra vulgaris* (Hydrozoa)

*X-squared = 299.26, df = 3, p-value < 2.2e-16*

|  | Low | High |
| --- | --- | --- |
| Low | 554 | 424 |
| High | 743 | 1027 |

*Alatina alata* (Cubozoa) *vs. Acropora digitifera* (Anthozoa)

*X-squared = 716.96, df = 3, p-value < 2.2e-16*

|  | Low | High |
| --- | --- | --- |
| Low | 344 | 575 |
| High | 401 | 1189 |

*Clytia hemisphaerica* (Hydrozoa) *vs. Aurelia coerulia* (Scyphozoa)

*X-squared = 73.083, df = 3, p-value = 9.329e-16*

|  | Low | High |
| --- | --- | --- |
| Low | 352 | 281 |
| High | 207 | 408 |

*Clytia hemisphaerica* (Hydrozoa) *vs. Nematostella vectensis* (Anthozoa)

*X-squared = 446.22, df = 3, p-value < 2.2e-16*

|  | Low | High |
| --- | --- | --- |
| Low | 186 | 404 |
| High | 109 | 593 |

*Clytia hemisphaerica* (Hydrozoa) *vs. Hydra vulgaris* (Hydrozoa)

*X-squared = 52.6, df = 3, p-value = 2.231e-11*

|  | Low | High |
| --- | --- | --- |
| Low | 433 | 320 |
| High | 341 | 500 |

*Clytia hemisphaerica* (Hydrozoa) *vs. Acropora digitifera* (Anthozoa)

*X-squared = 218.96, df = 3, p-value < 2.2e-16*

|  | Low | High |
| --- | --- | --- |
| Low | 239 | 395 |
| High | 193 | 540 |

*Clytia hemisphaerica* (Hydrozoa) *vs. Alatina alata* (Cubozoa)

*X-squared = 63.043, df = 3, p-value = 1.315e-13*

|  | Low | High |
| --- | --- | --- |
| Low | 366 | 387 |
| High | 282 | 500 |

*Physalia physalis* (Hydrozoa) *vs. Aurelia coerulia* (Scyphozoa)

*X-squared = 54.11, df = 3, p-value = 1.063e-11*

|  | Low | High |
| --- | --- | --- |
| Low | 313 | 293 |
| High | 310 | 462 |

*Physalia physalis* (Hydrozoa) *vs. Nematostella vectensis* (Anthozoa)

*X-squared = 562.62, df = 3, p-value < 2.2e-16*

|  | Low | High |
| --- | --- | --- |
| Low | 150 | 433 |
| High | 131 | 665 |

*Physalia physalis* (Hydrozoa) *vs. Hydra vulgaris* (Hydrozoa)

*X-squared = 123.3, df = 3, p-value< 2.2e-16*

|  | Low | High |
| --- | --- | --- |
| Low | 486 | 300 |
| High | 461 | 628 |

*Physalia physalis* (Hydrozoa) *vs. Acropora digitifera* (Anthozoa)

*X-squared = 317.6, df = 3, p-value < 2.2e-16*

|  | Low | High |
| --- | --- | --- |
| Low | 221 | 376 |
| High | 225 | 640 |

*Physalia physalis* (Hydrozoa) *vs. Alatina alata* (Cubozoa)

*X-squared = 113.57, df = 3, p-value = 1.315e-13*

|  | Low | High |
| --- | --- | --- |
| Low | 358 | 373 |
| High | 307 | 611 |

*Physalia physalis* (Hydrozoa) *vs. Clytia hemisphaerica* (Hydrozoa)

*X-squared = 42.297, df = 3, p-value = 2.854e-09*

|  | Low | High |
| --- | --- | --- |
| Low | 327 | 242 |
| High | 281 | 397 |

Fig. S1. Density distribution of gene body CpG_o/e_ of the 76 species in this study. For each species, the left panel shows the density distribution and mode detection by Notos. The blue or orange vertical lines denote the peaks of modes detected. 11 species are detected to be bimodal. The middle panel shows BIC scores of Gaussian mixture models. Most species are best described by mixture models with three or more components. The right panel shows correlation between CpG_o/e_ and TpG_o/e_ to verify that the depletion of CpG is indeed due to cytosine methylation.

Fig. S2. Phylogenetic analyses of DNMT1 and DNMT3. Sequences found in *Myxobolus pendula* are more closely related to corresponding sequences found in the zebrafish than the other Cnidarians.


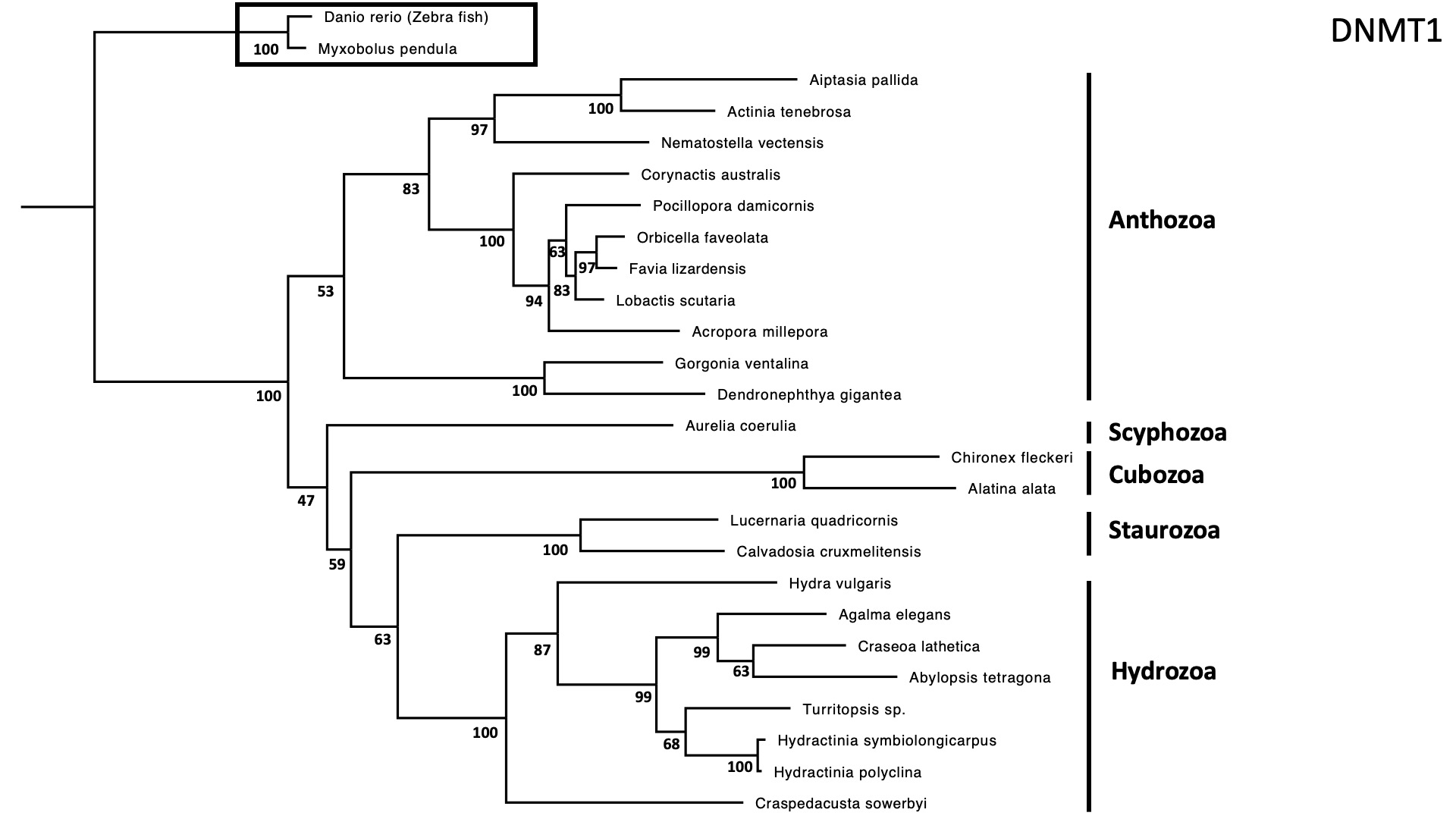


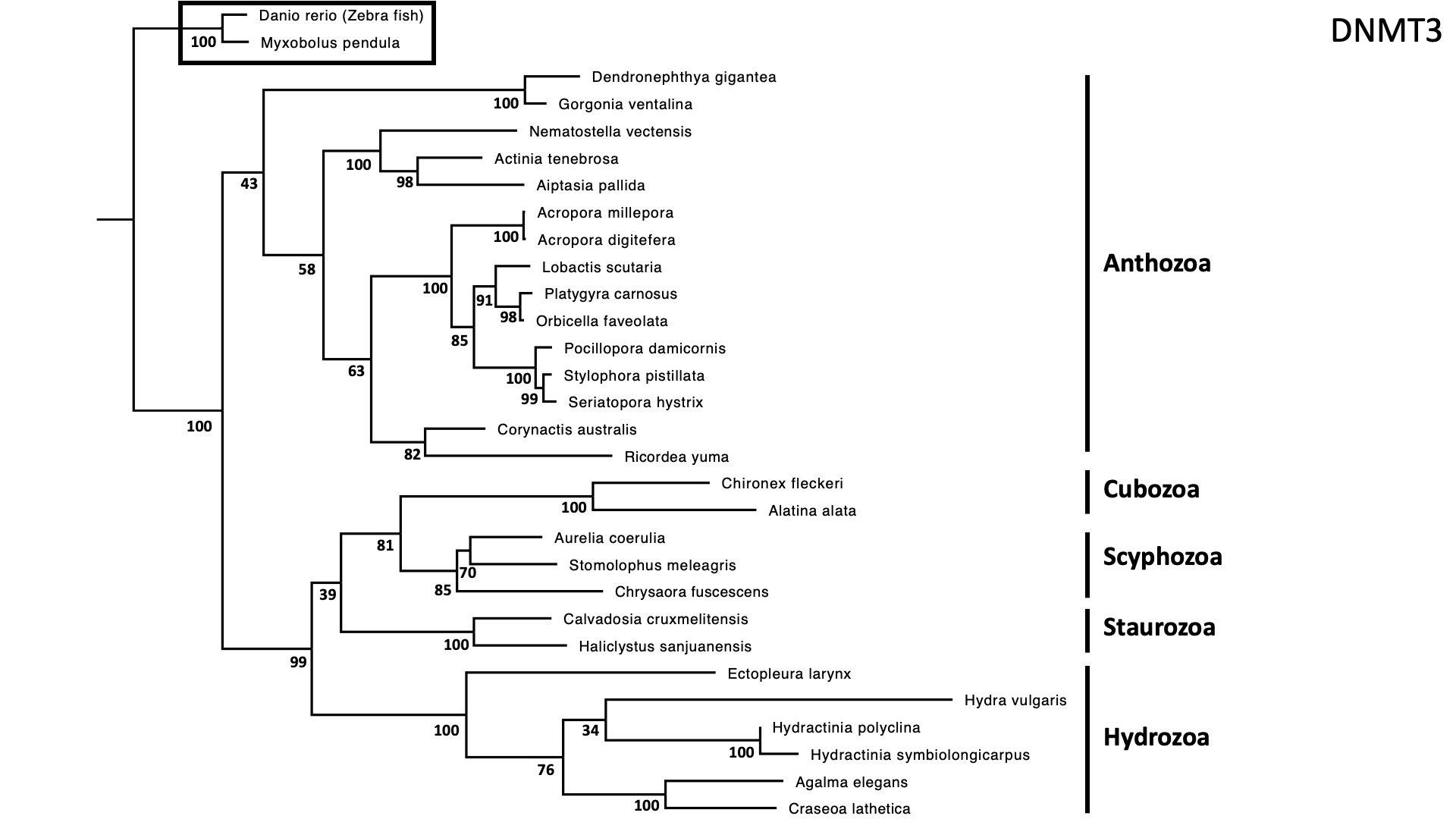


### **Supplemental References:**

Brekhman V, Malik A, Haas B, Sher N, Lotan T. 2015. Transcriptome profiling of the dynamic life cycle of the scypohozoan jellyfish Aurelia aurita. *BMC genomics* 16:74.

Hasegawa Y, Watanabe T, Takazawa M, Ohara O, Kubota S. 2016. De novo assembly of the transcriptome of Turritopsis, a jellyfish that repeatedly rejuvenates. *Zoological science* 33:366–371.

Okubo N, Hayward DC, Forêt S, Ball EE. 2016. A comparative view of early development in the corals Favia lizardensis, Ctenactis echinata, and Acropora millepora-morphology, transcriptome, and developmental gene expression. *BMC evolutionary biology* 16:48.

Stefanik DJ, Lubinski TJ, Granger BR, Byrd AL, Reitzel AM, DeFilippo L, Lorenc A, Finnerty JR. 2014. Production of a reference transcriptome and transcriptomic database (EdwardsiellaBase) for the lined sea anemone, Edwardsiella lineata, a parasitic cnidarian. *BMC genomics* 15:71.
