# Supplementary material for: A Broad Survey of Gene Body and Repeat Methylation in Cnidaria Reveals a Complex Evolutionary History
Source: Genome Biol Evol. 2022 Feb 1;14(2):evab284. doi: 10.1093/gbe/evab284 (PMC8857923; doi:10.1093/gbe/evab284)
Supplement: evab284_Supplementary_Data [file evab284_supplementary_data.zip › FigS1_GBE_revised.pdf]

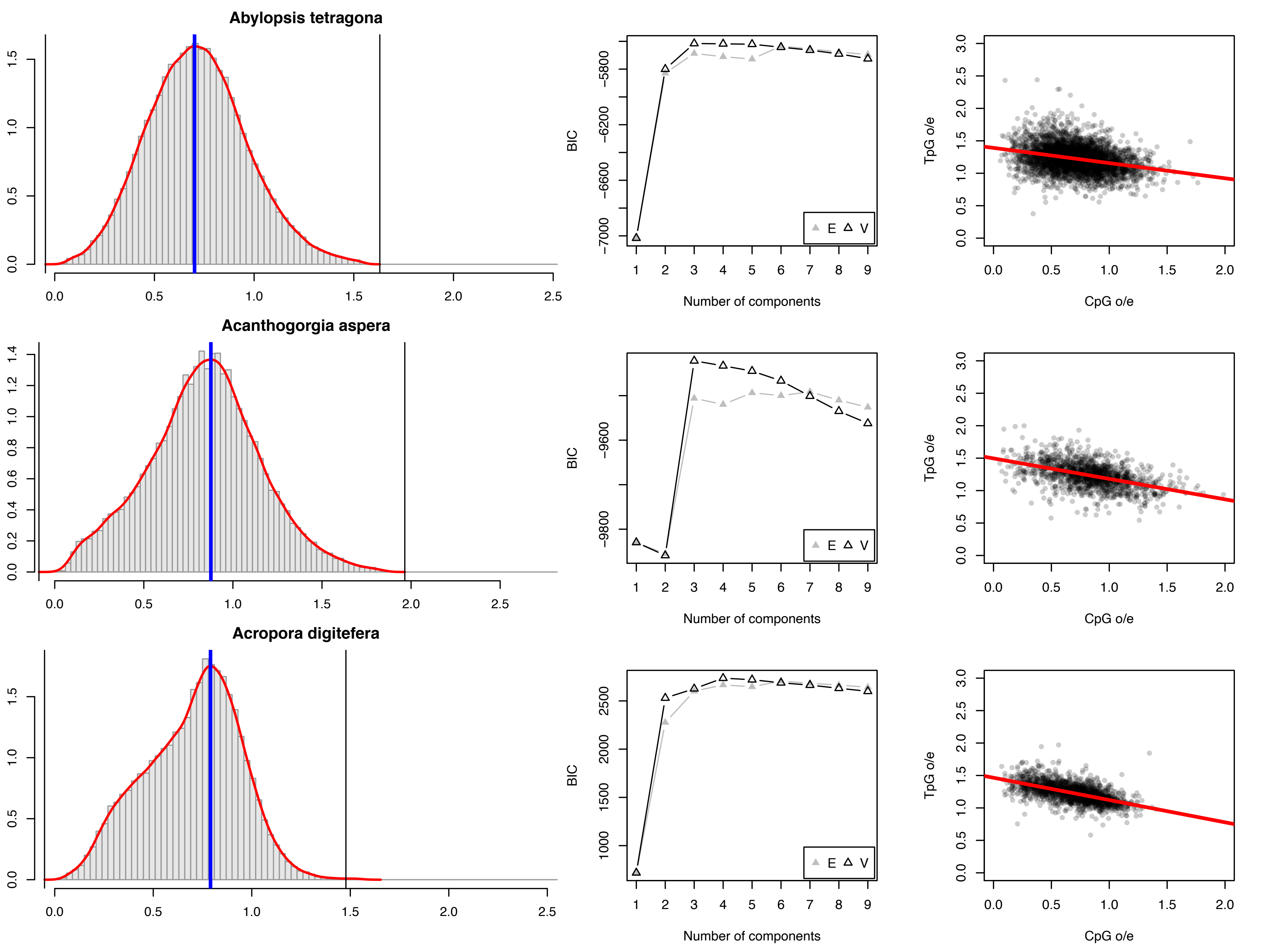

Abylopsis tetragona
Acanthogorgia aspera
Acropora digitefera
BIC
Number of components
E V
TpG o/e
CpG o/e

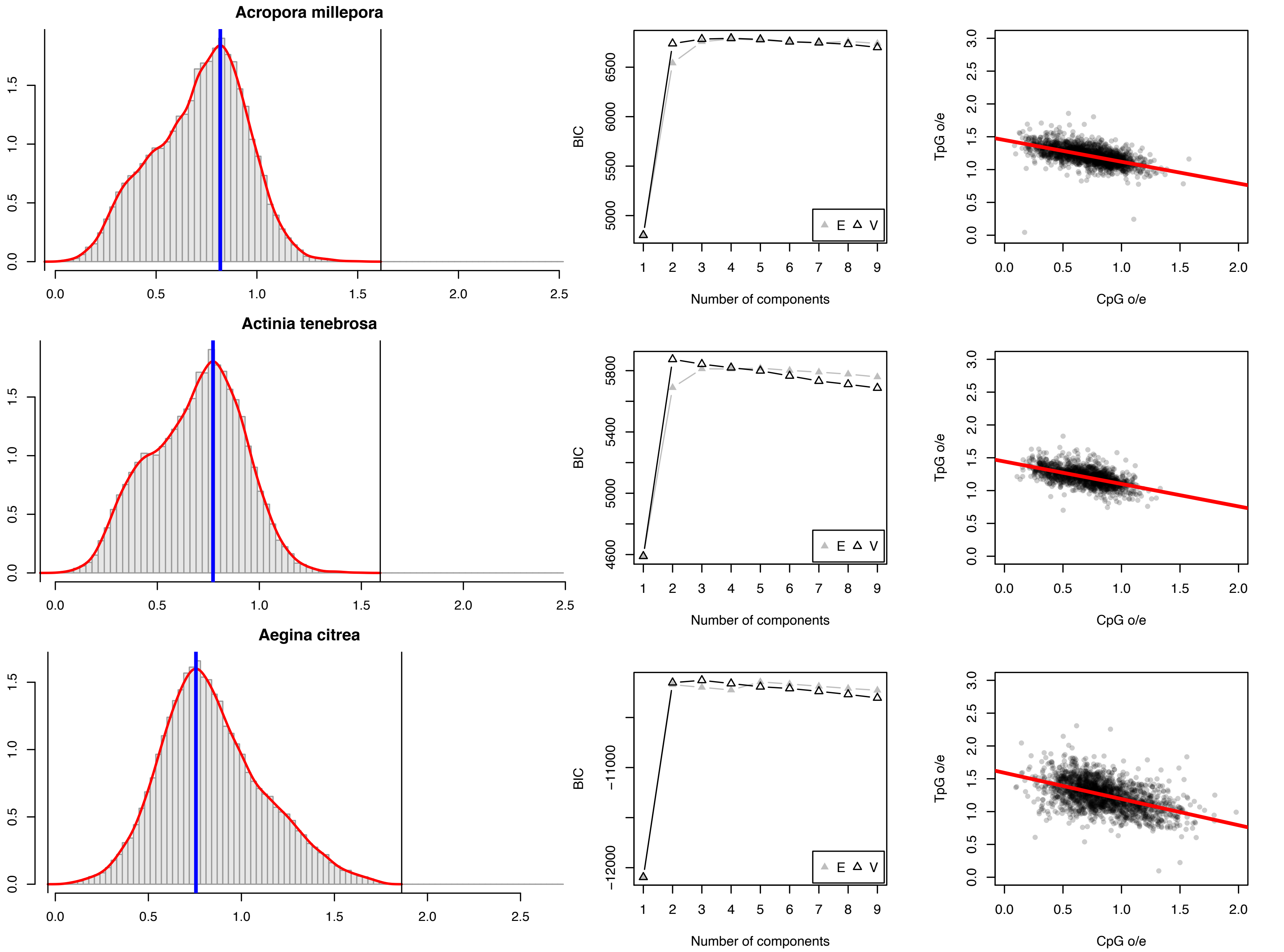

Acropora millepora
Actinia tenebrosa
Aegina citrea
BIC
Number of components
E
V
TpG o/e
CpG o/e
0.0
0.5
1.0
1.5
2.0
2.5
3.0
1
2
3
4
5
6
7
8
9
5000
5500
6000
6500
4600
5000
5400
5800
−12000
−11000

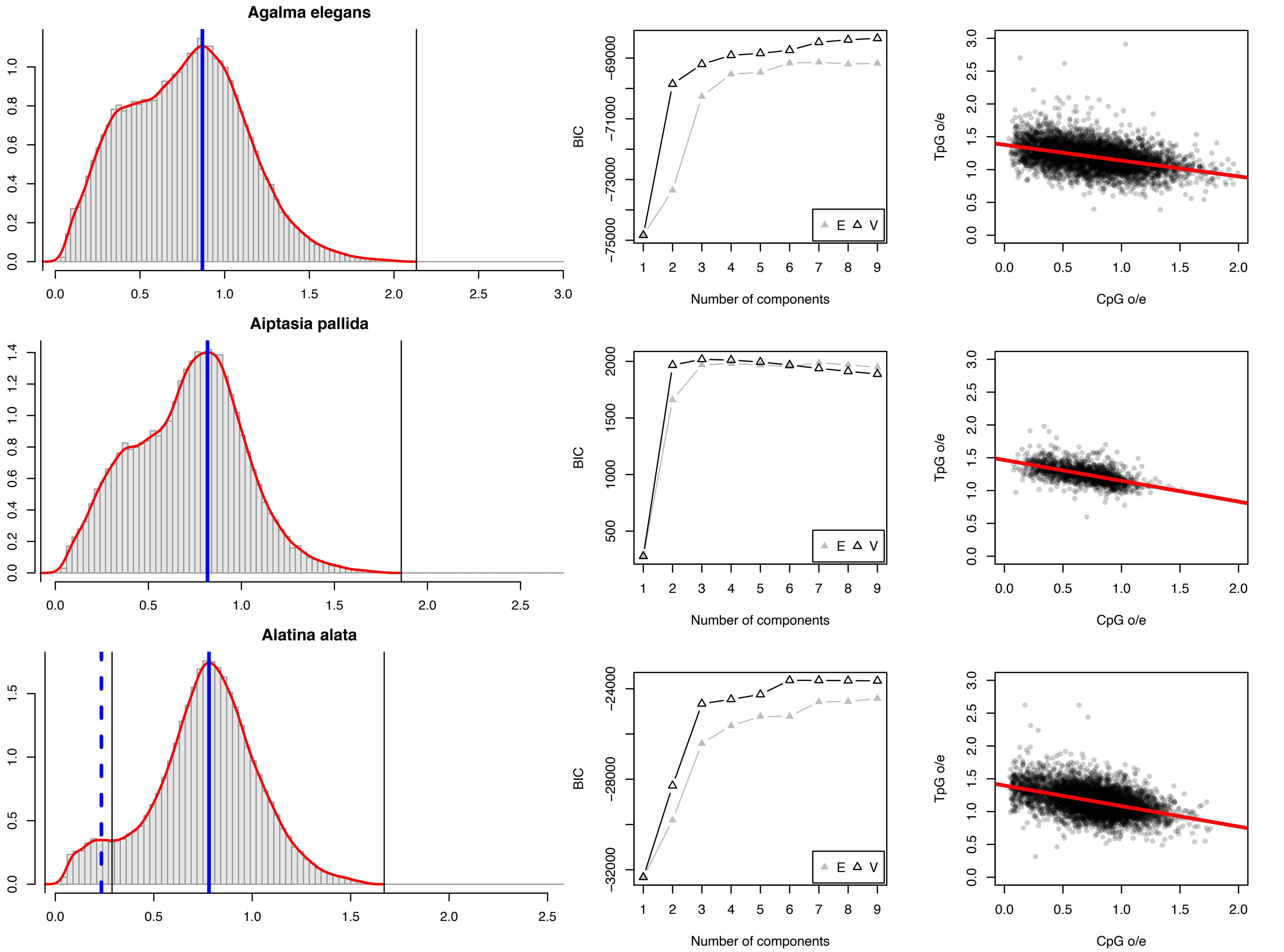

Agalma elegans
Aiptasia pallida
Alatina alata
BIC
Number of components
E
V
TpG o/e
CpG o/e
0.0
0.5
1.0
1.5
2.0
2.5
3.0
−69000
−71000
−73000
−75000
500
1000
1500
2000
−24000
−28000
−32000
1
2
3
4
5
6
7
8
9

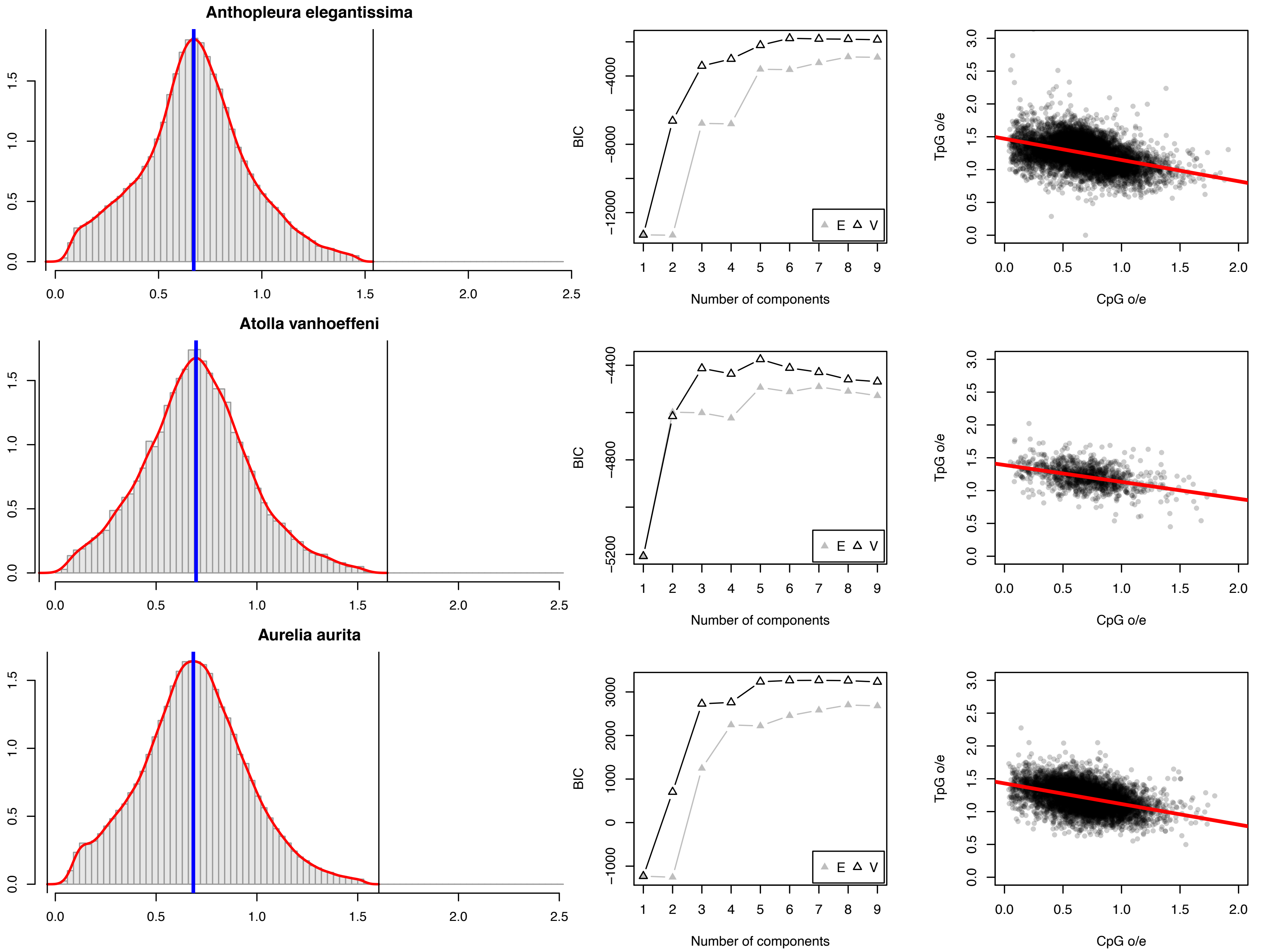

Anthopleura elegantissima
Atolla vanhoeffeni
Aurelia aurita
BIC
Number of components
E
V
TpG o/e
CpG o/e

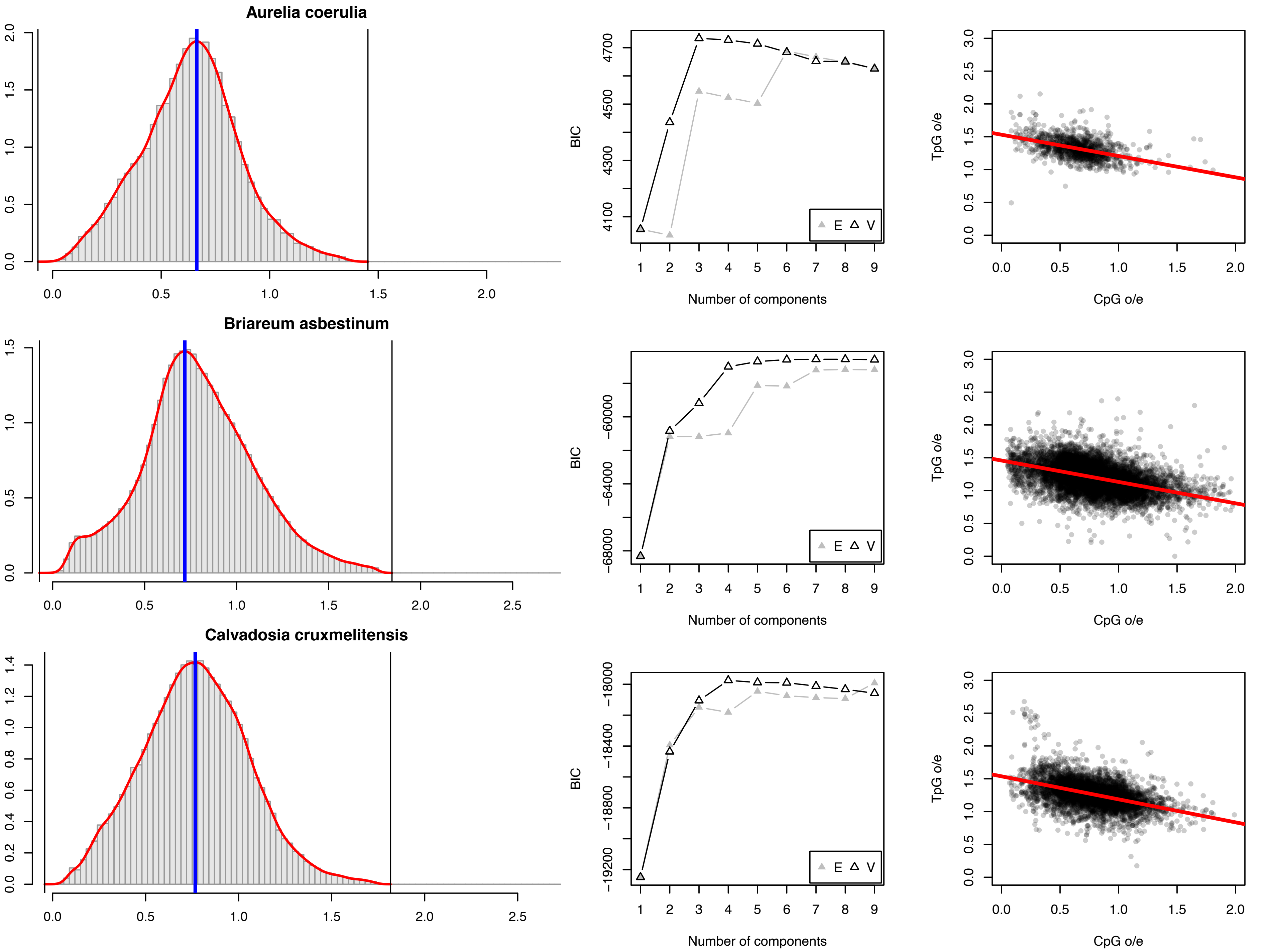
Aurelia coerulia
Briareum asbestinum
Calvadosia cruxmelitensis
BIC
Number of components
E
V
TpG o/e
CpG o/e

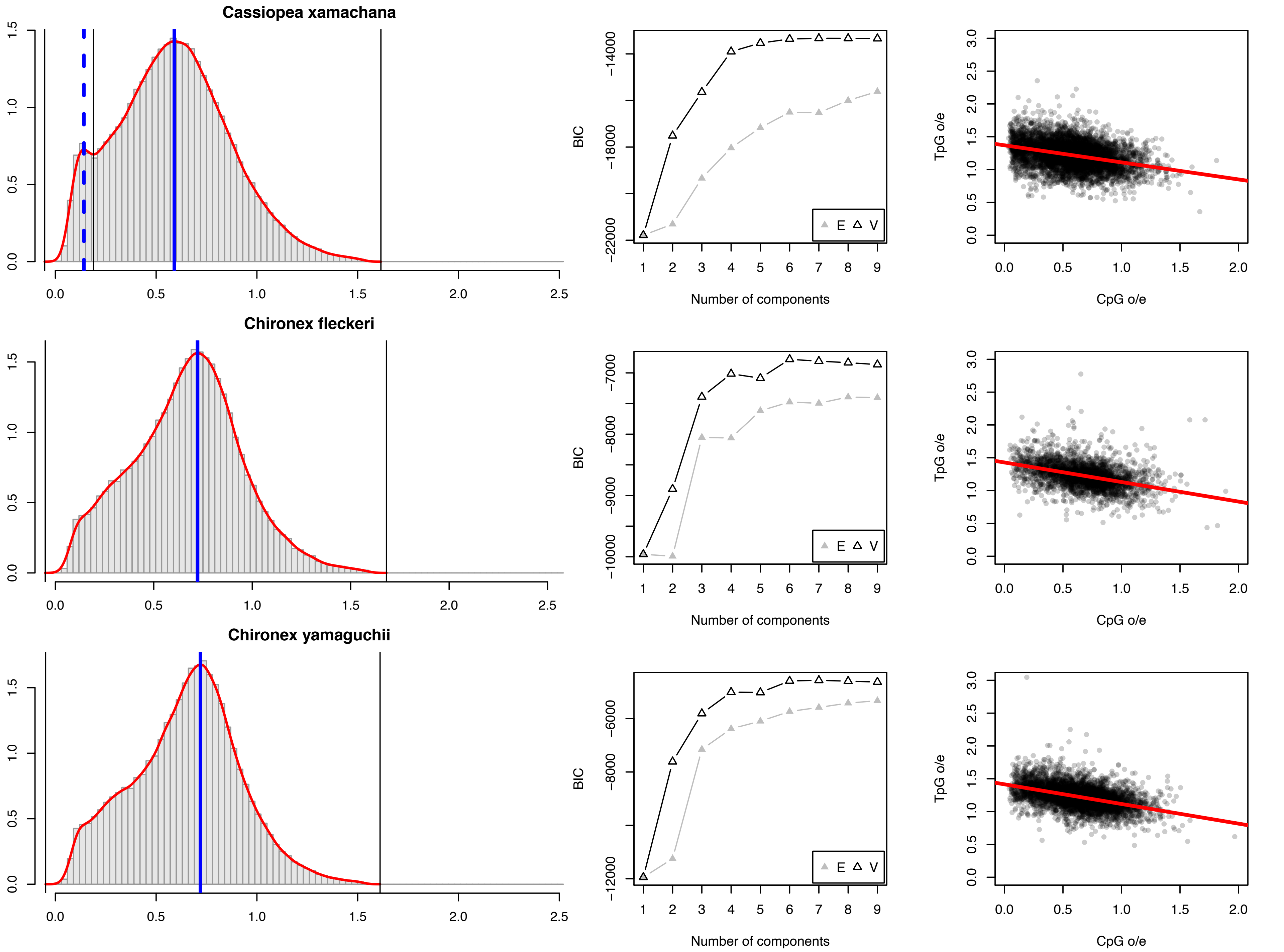

Cassiopea xamachana
Chironex fleckeri
Chironex yamaguchii
BIC
Number of components
E
V
TpG o/e
CpG o/e

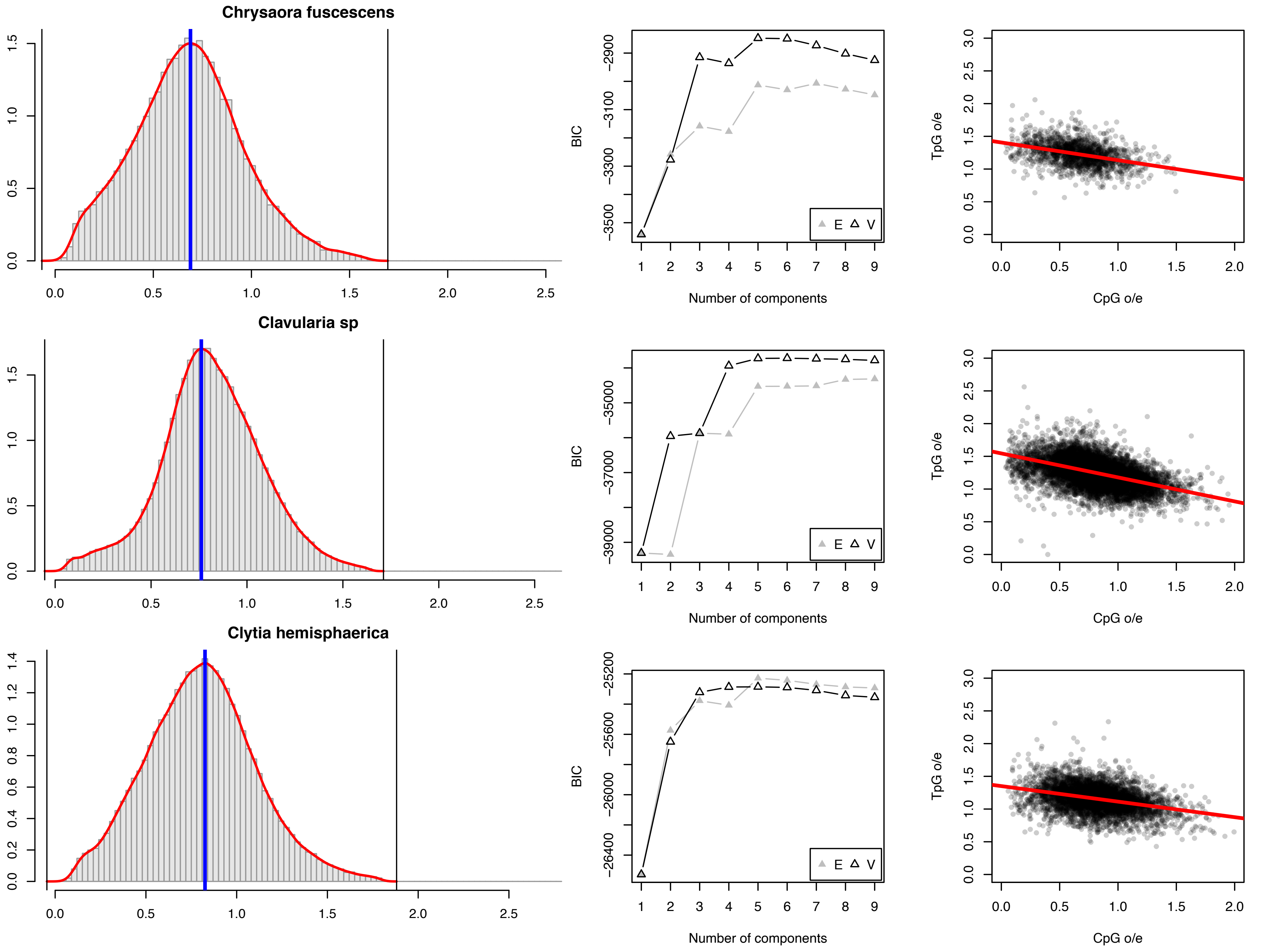

Chrysaora fuscescens
Clavularia sp
Clytia hemisphaerica
BIC
Number of components
E
V
TpG o/e
CpG o/e

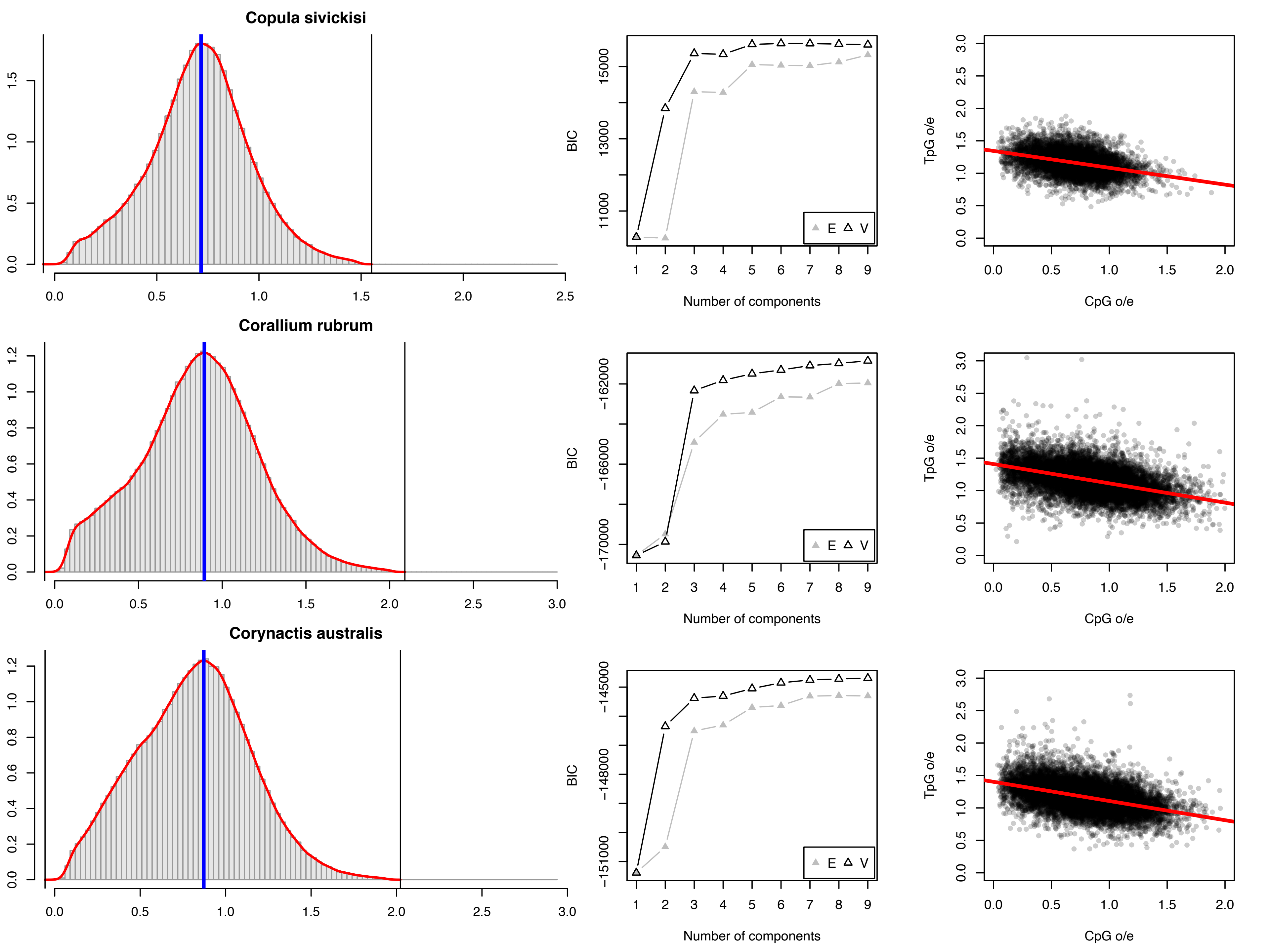
Copula sivickisi
Corallium rubrum
Corynactis australis
BIC
Number of components
E
V
TpG o/e
CpG o/e

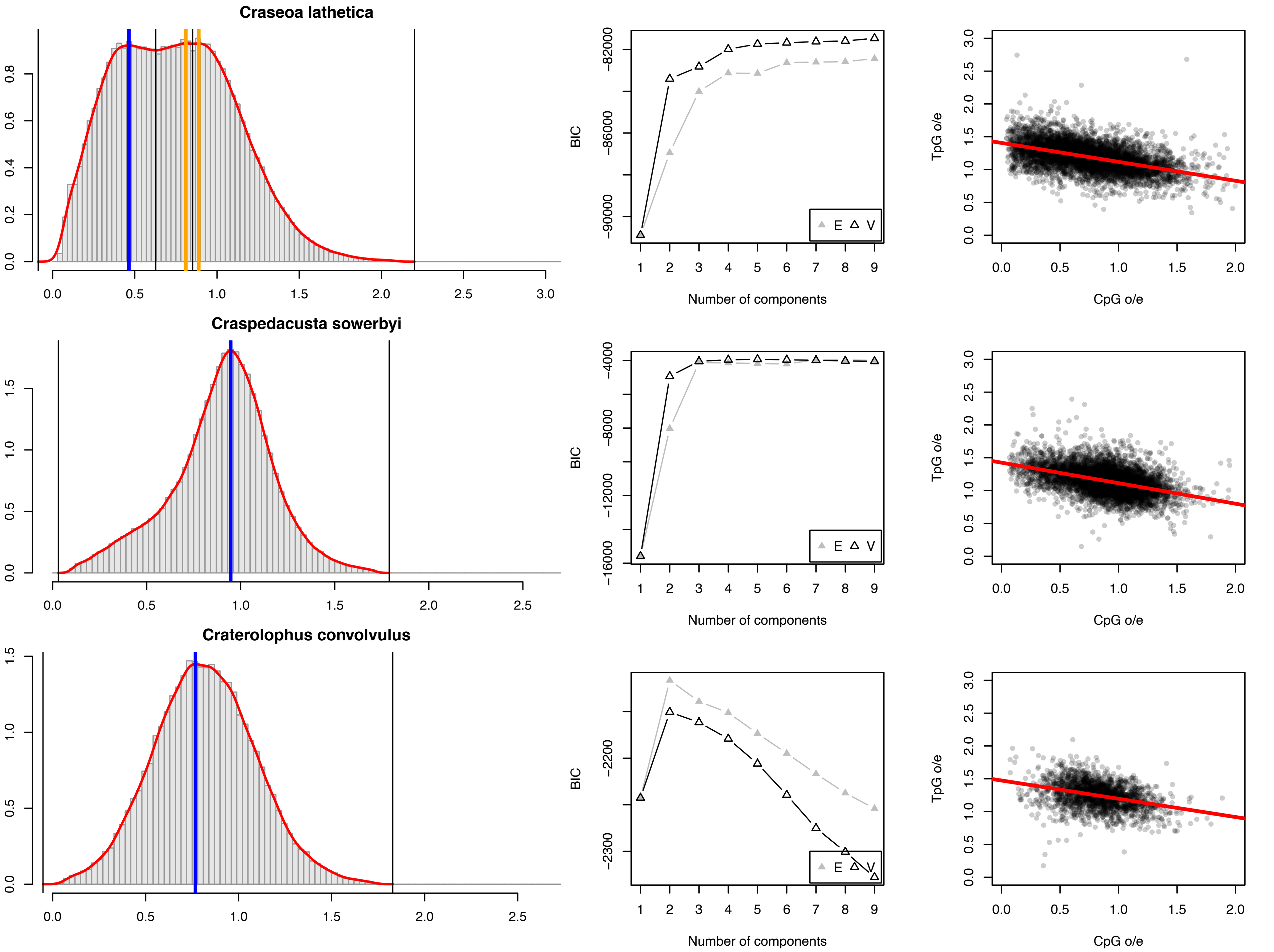

Craseoa lathetica
Craspedacusta sowerbyi
Craterolophus convolvulus
BIC
Number of components
E
V
TpG o/e
CpG o/e

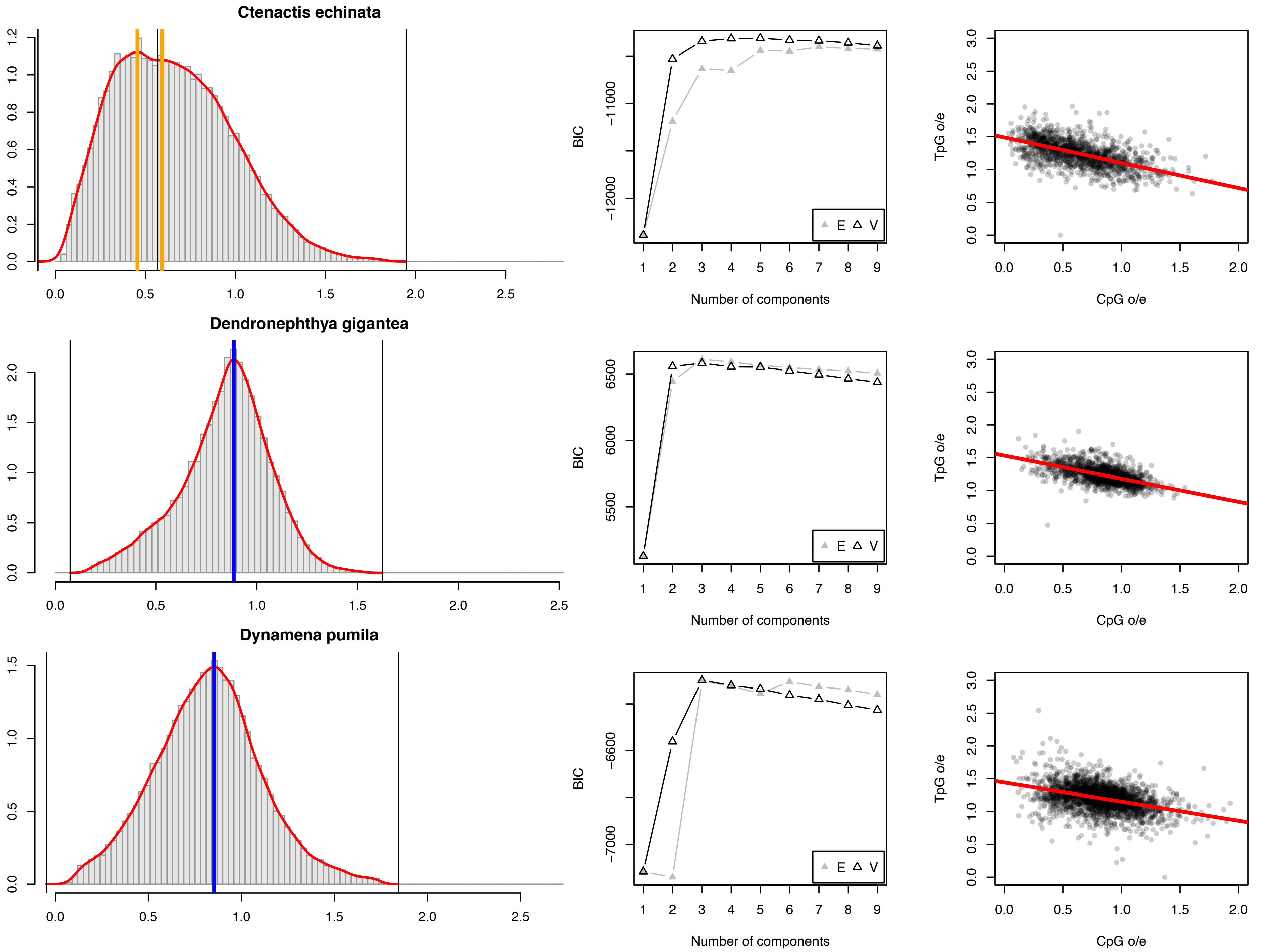

Ctenactis echinata
Dendronephthya gigantea
Dynamena pumila
BIC
Number of components
E
V
TpG o/e
CpG o/e

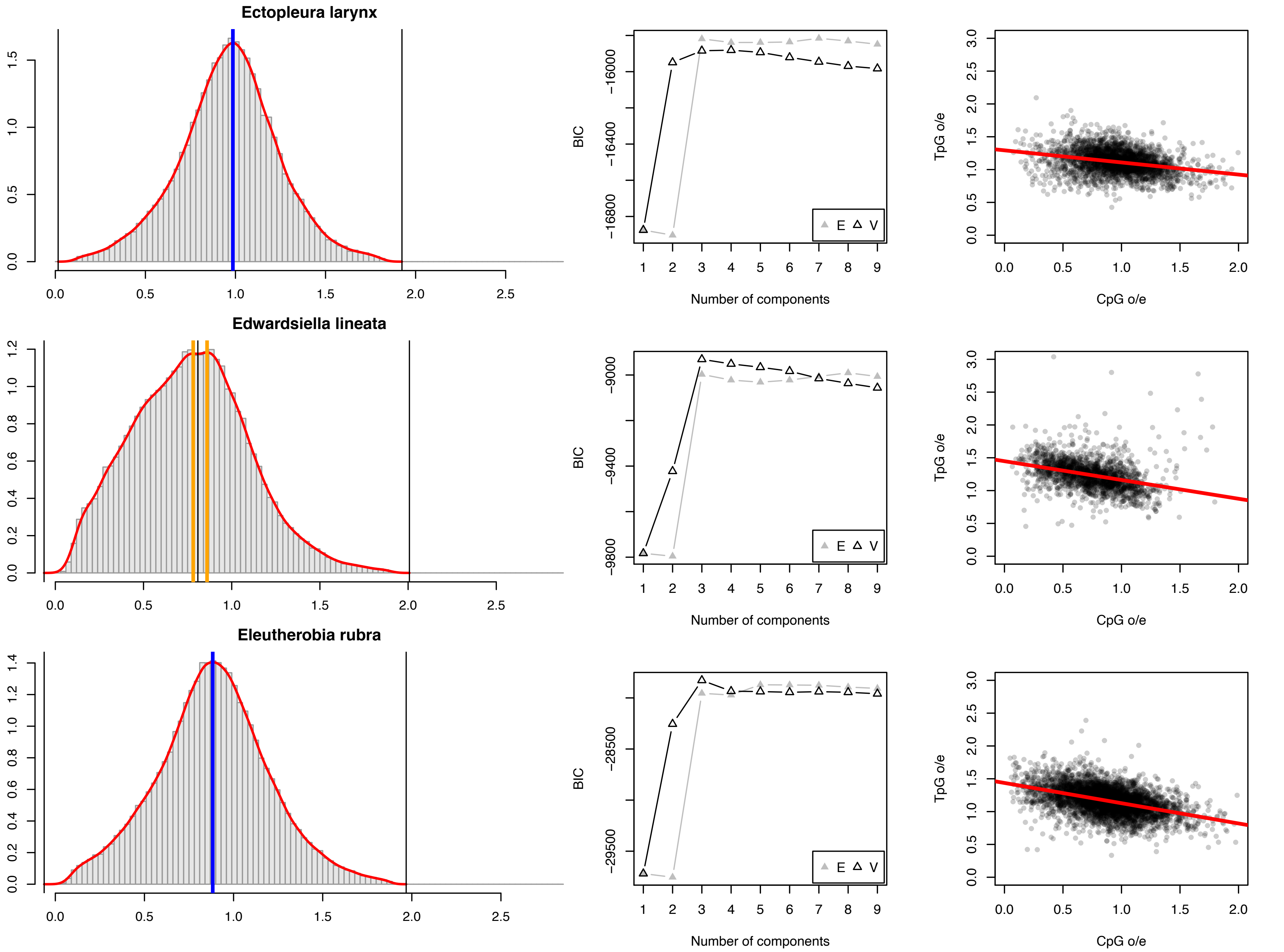
Ectopleura larynx
Edwardsiella lineata
Eleutherobia rubra
BIC
Number of components
E
V
TpG o/e
CpG o/e
−16000
−16400
−16800
−9000
−9400
−9800
−28500
−29500

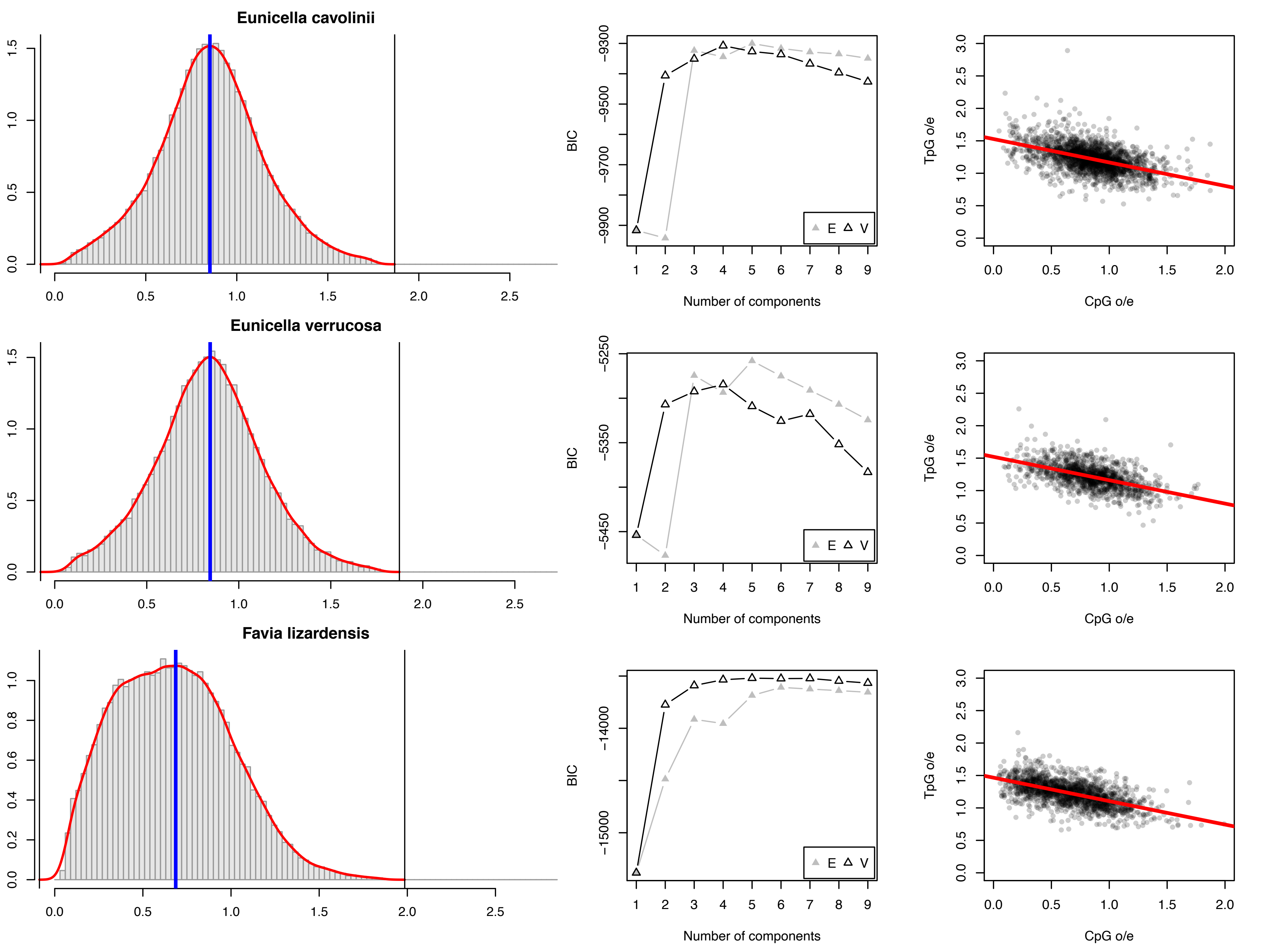
Eunicella cavolinii
Eunicella verrucosa
Favia lizardensis
BIC
Number of components
E
V
TpG o/e
CpG o/e
-9300
-9500
-9700
-9900
-5250
-5350
-5450
-14000
-15000
0.0
0.5
1.0
1.5
2.0
2.5
3.0
1
2
3
4
5
6
7
8
9

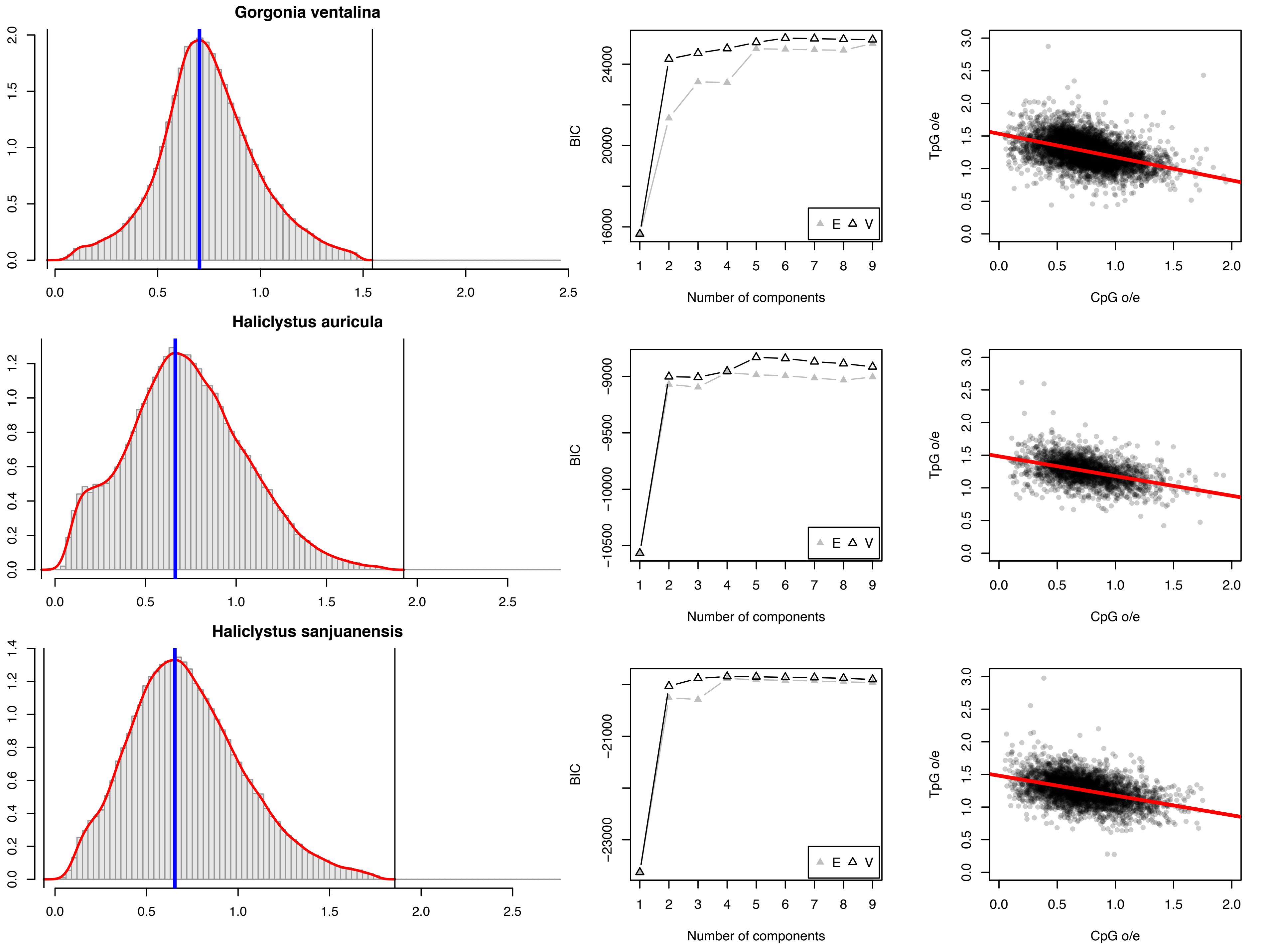
Gorgonia ventalina
Haliclystus auricula
Haliclystus sanjuanensis
BIC
Number of components
E V
TpG o/e
CpG o/e

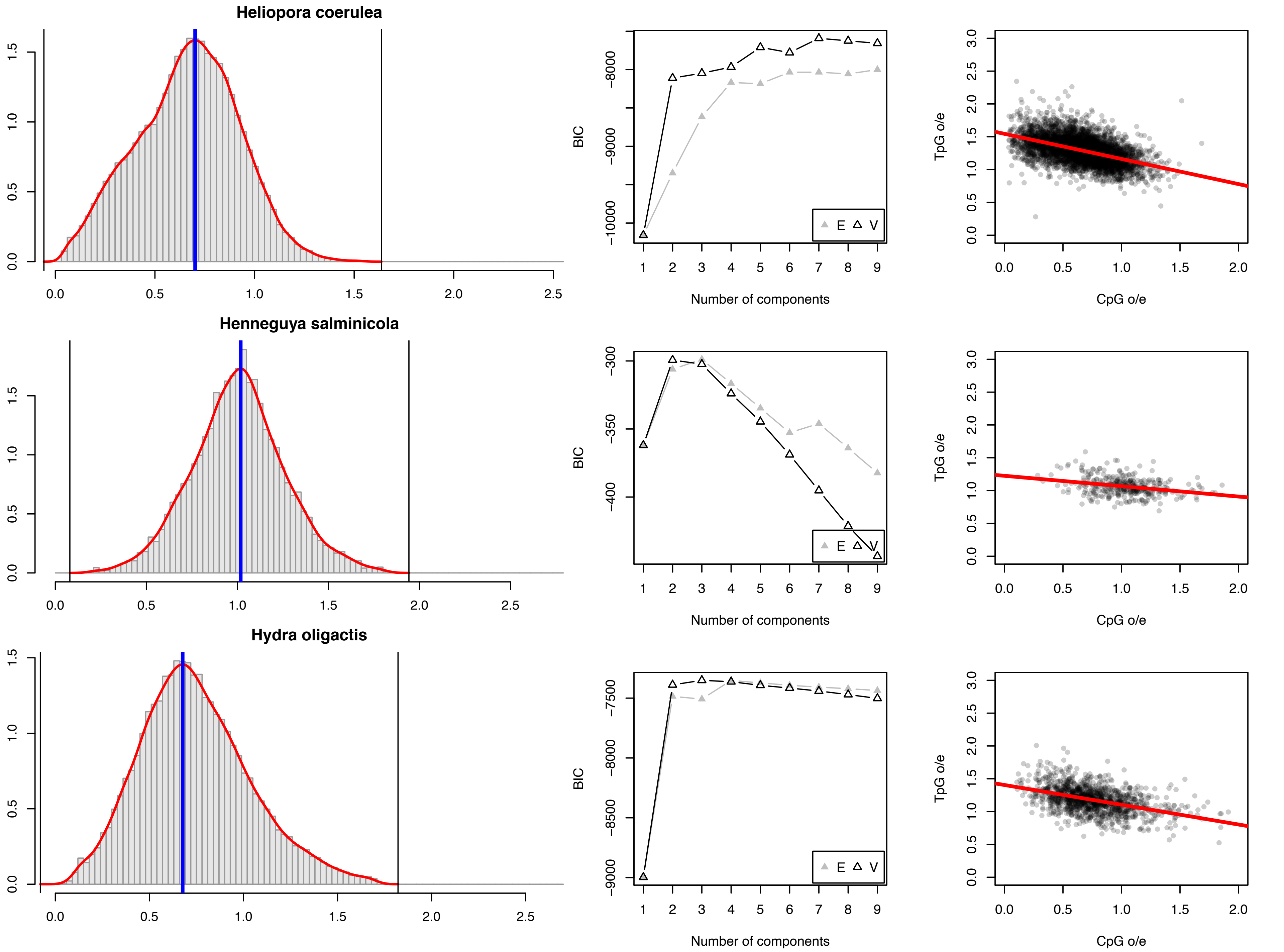
Heliopora coerulea
Henneguya salminicola
Hydra oligactis
BIC
Number of components
E V
TpG o/e
CpG o/e

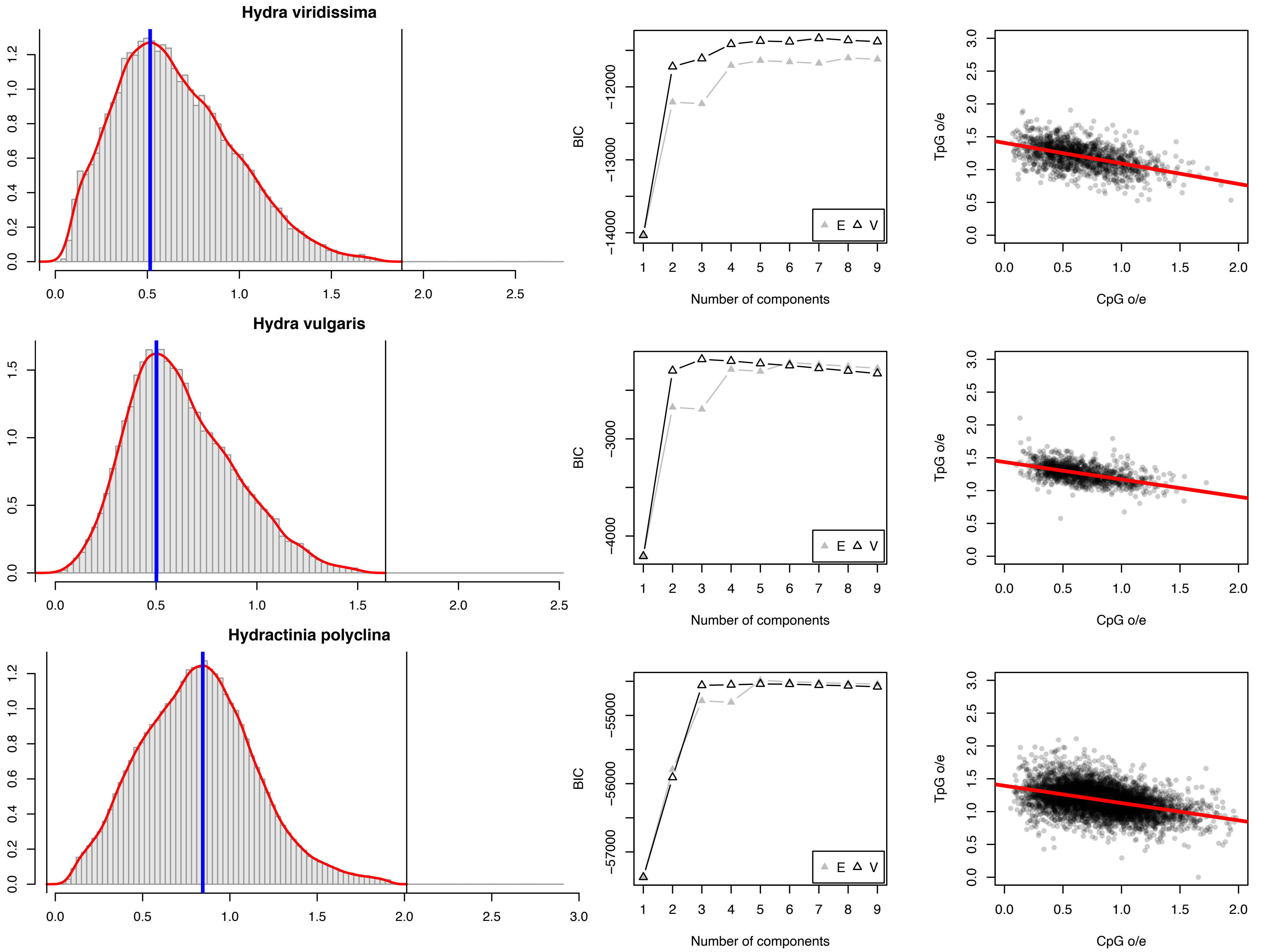

Hydra viridissima
Hydra vulgaris
Hydractinia polyclina
BIC
Number of components
E
V
TpG o/e
CpG o/e

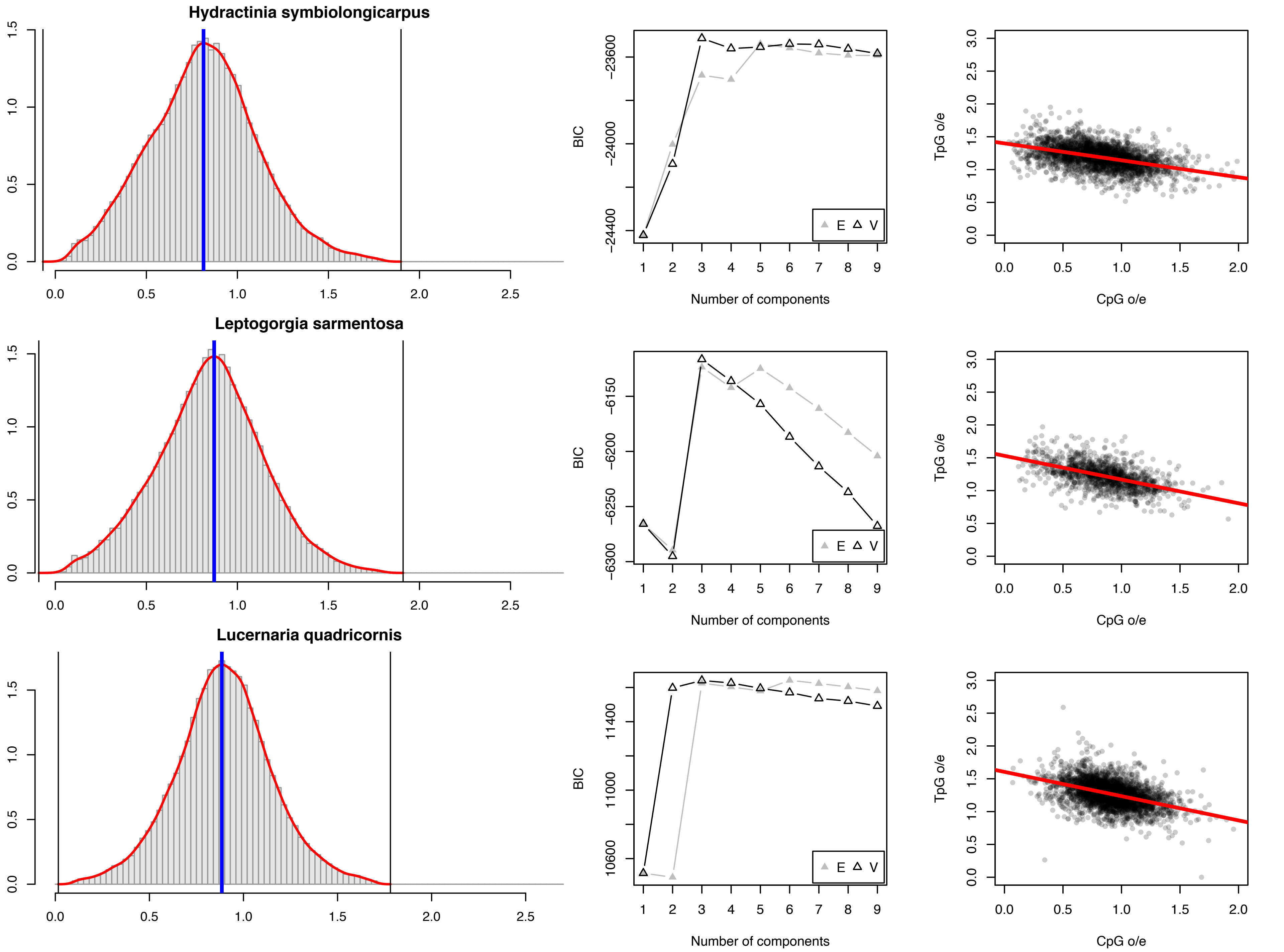
Hydractinia symbiolongicarpus
Leptogorgia sarmentosa
Lucernaria quadricornis
BIC
Number of components
E
V
TpG o/e
CpG o/e

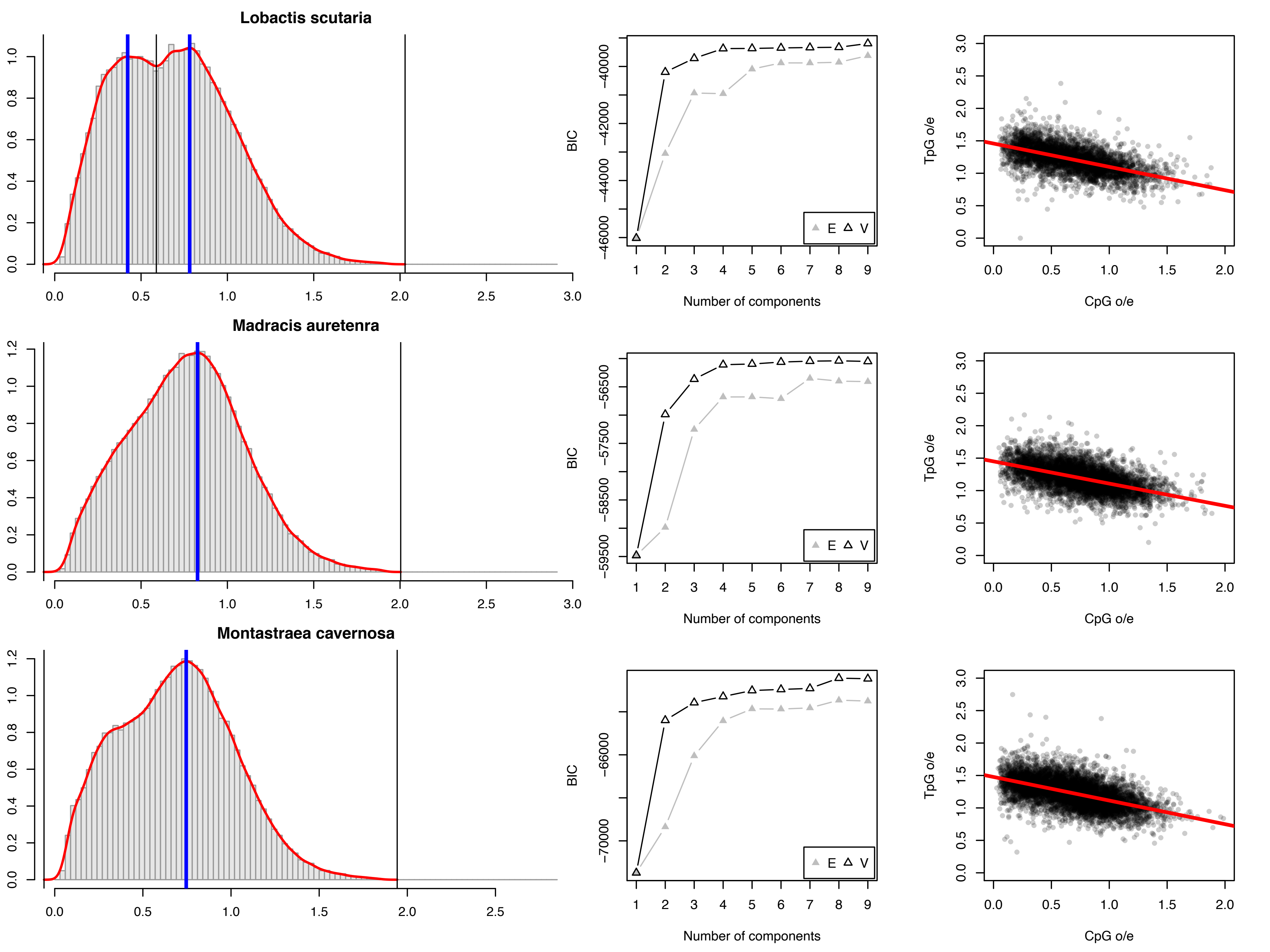

Lobactis scutaria
Madracis auretenra
Montastraea cavernosa
BIC
Number of components
E
V
TpG o/e
CpG o/e

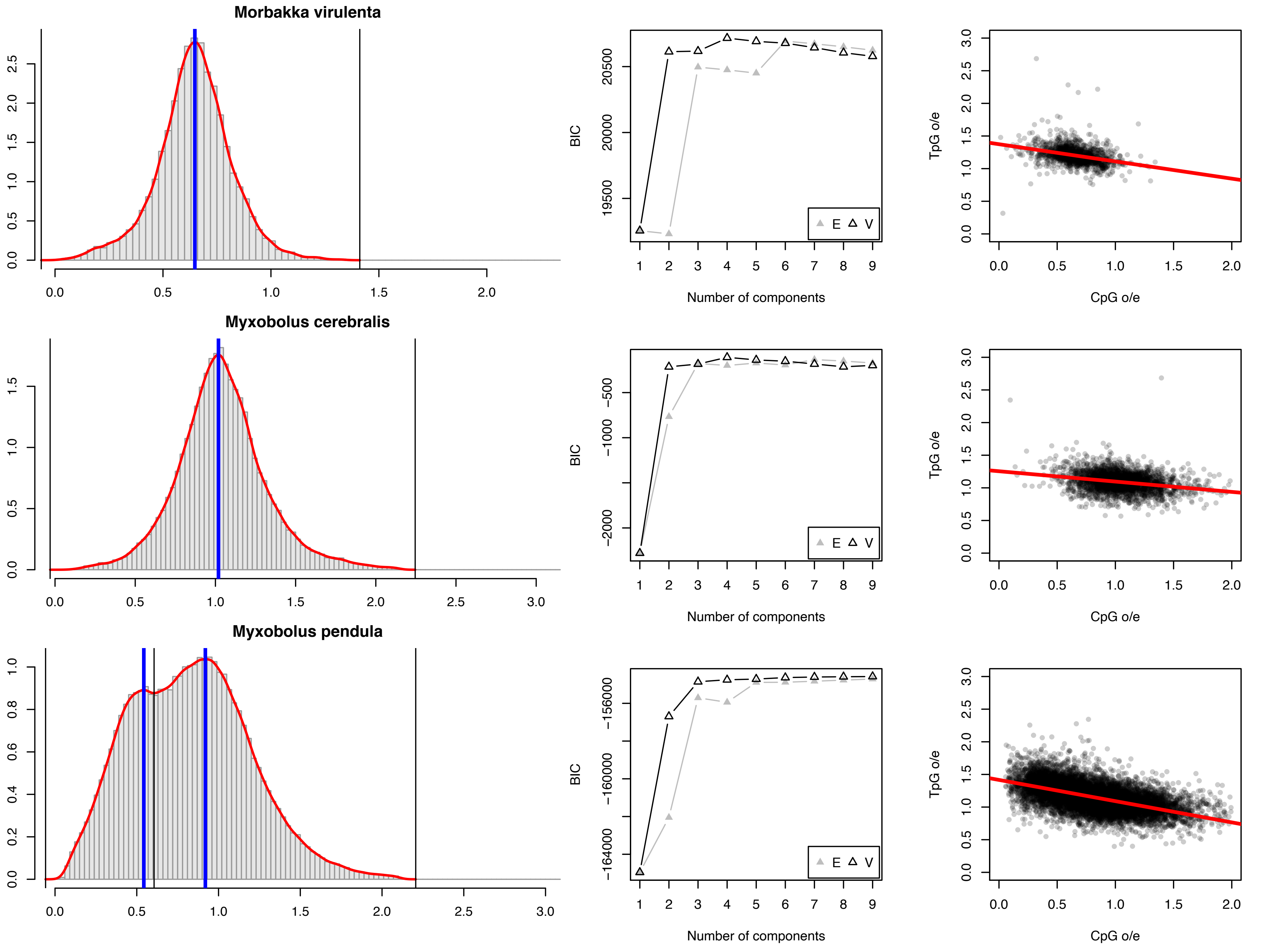

Morbakka virulenta
Myxobolus cerebralis
Myxobolus pendula
BIC
Number of components
E
V
TpG o/e
CpG o/e

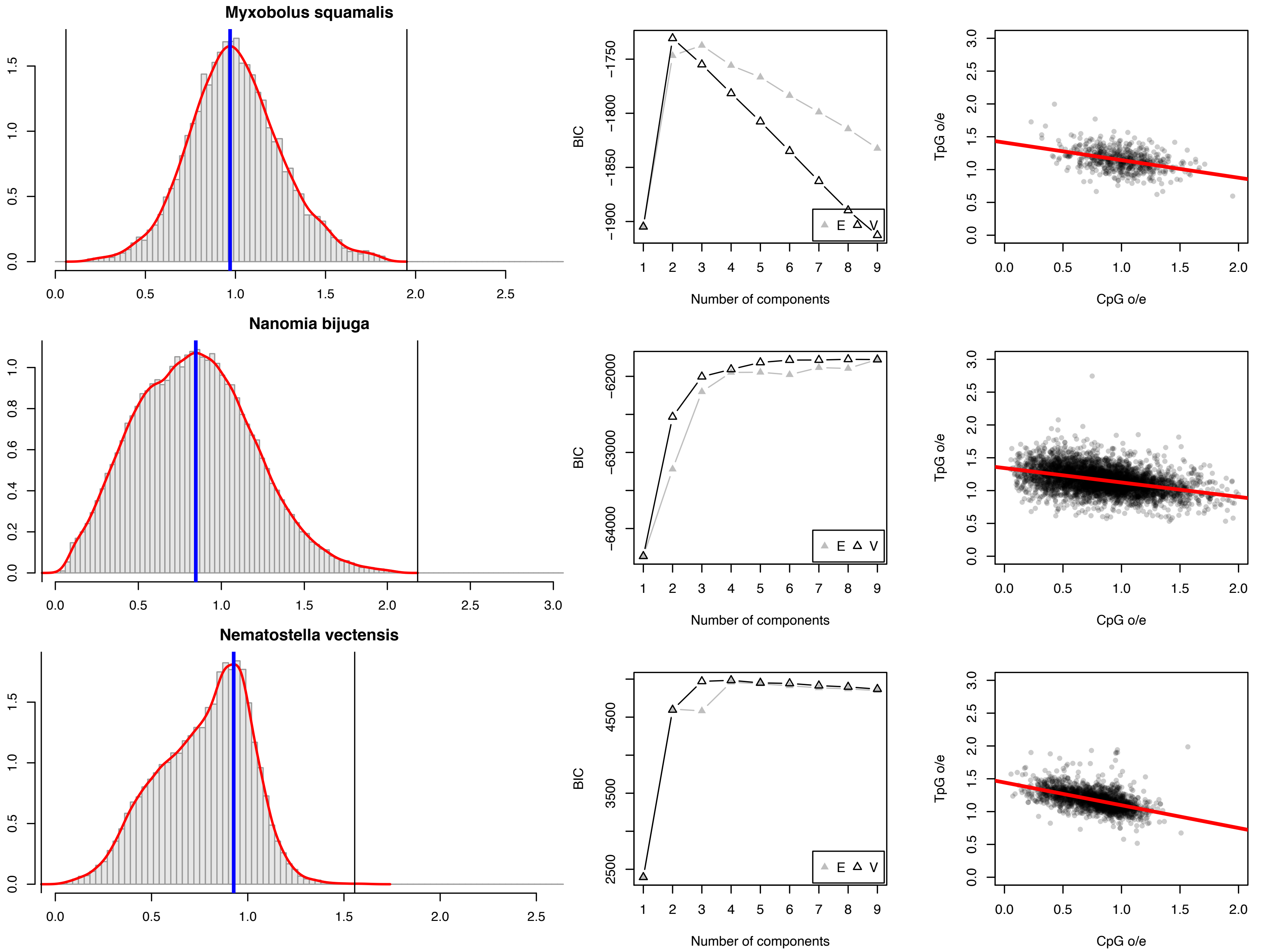

Myxobolus squamalis
Nanomia bijuga
Nematostella vectensis
BIC
Number of components
E
V
TpG o/e
CpG o/e

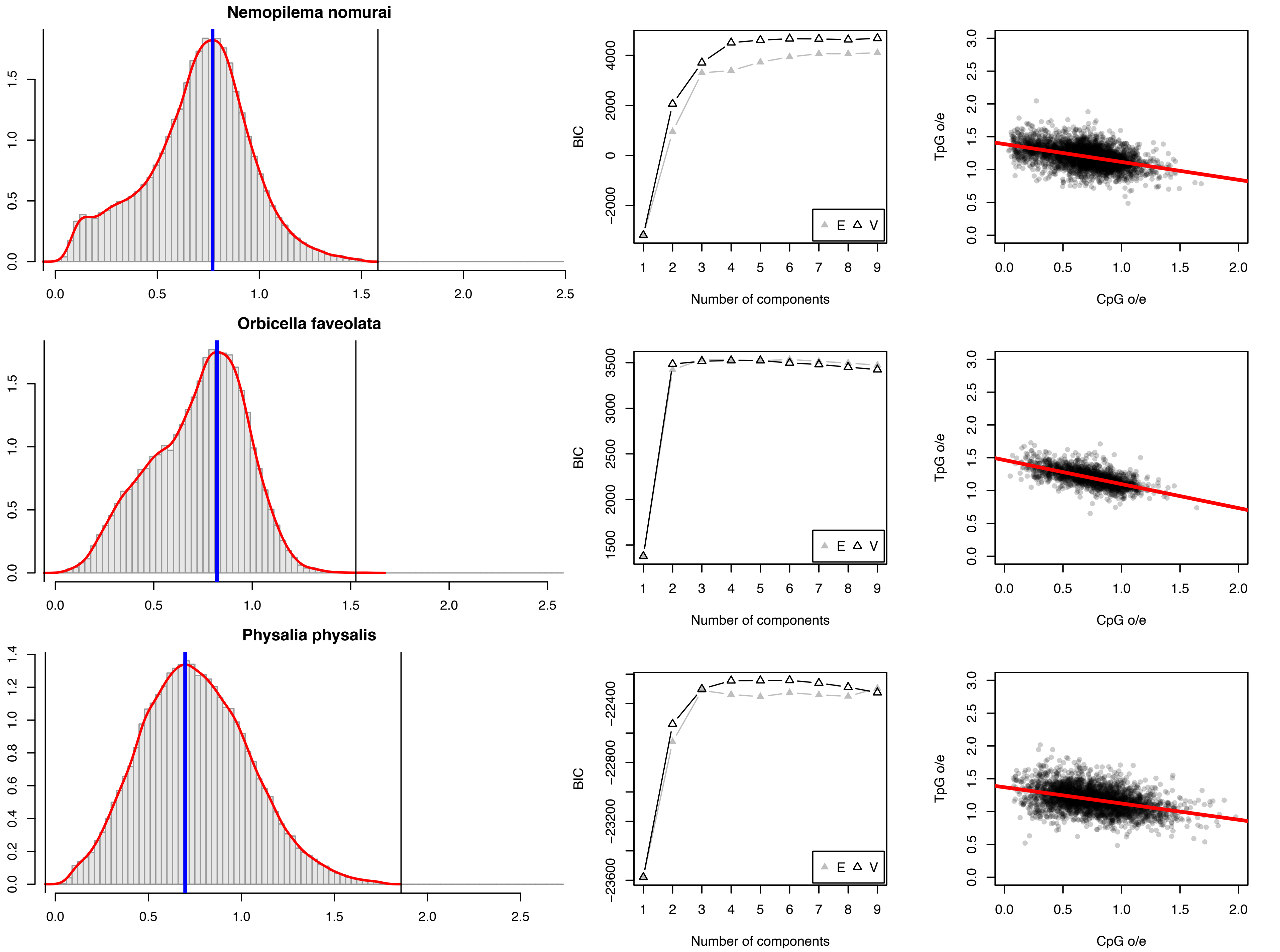
Nemopilema nomurai
Orbicella faveolata
Physalia physalis
BIC
Number of components
E
V
TpG o/e
CpG o/e

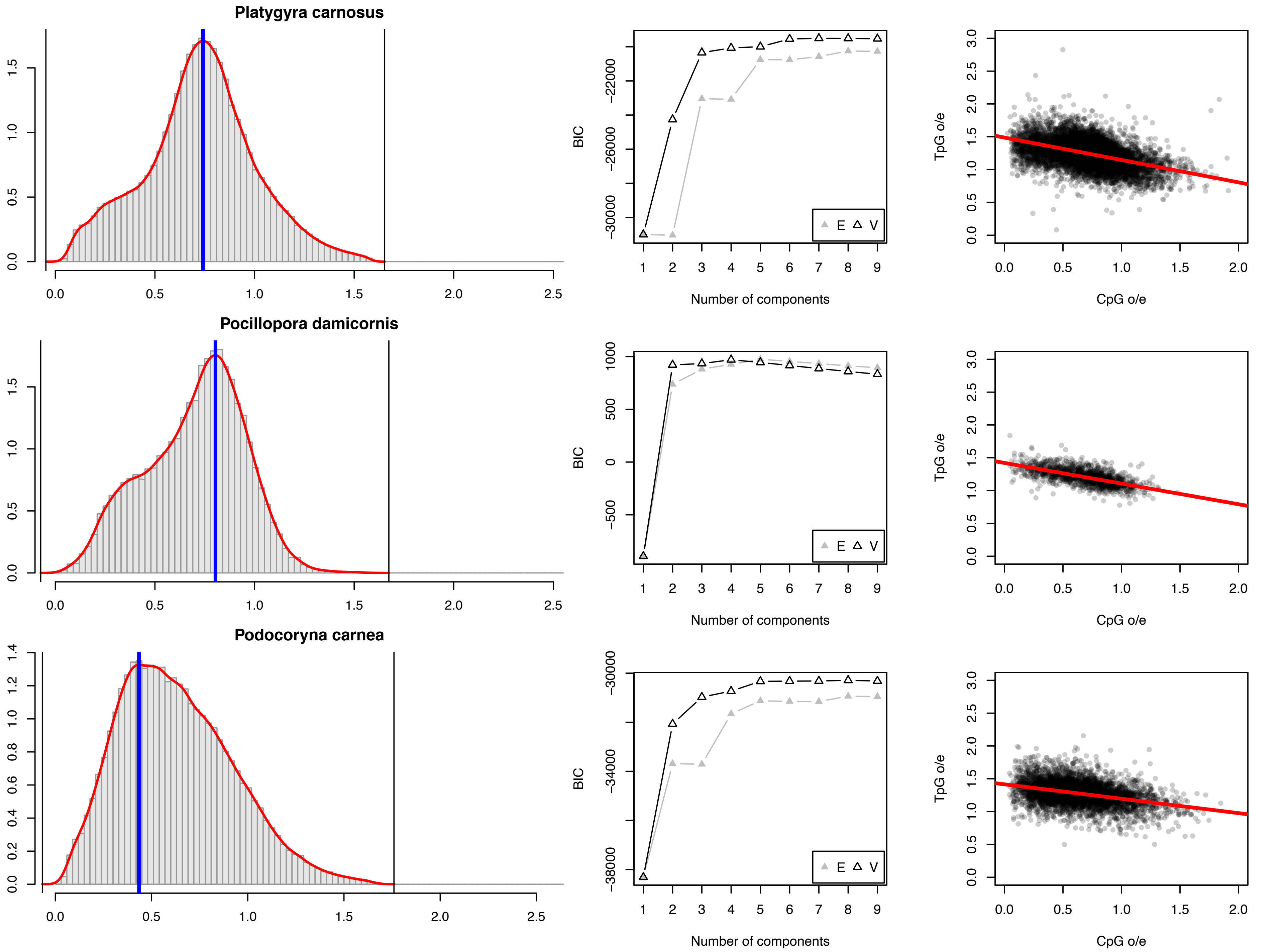

Platygyra carnosus
Pocillopora damicornis
Podocoryna carnea
BIC
Number of components
E
V
TpG o/e
CpG o/e

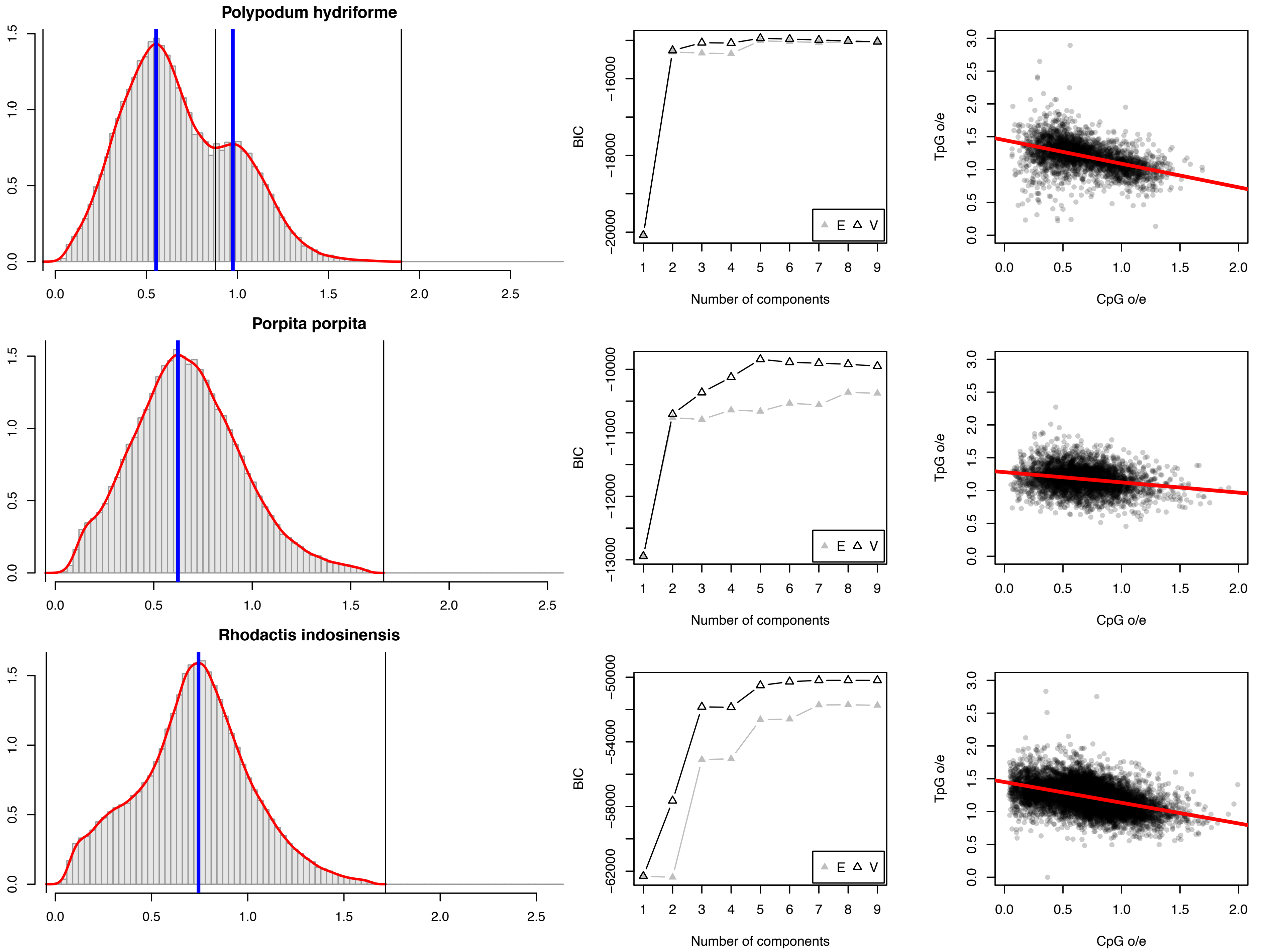

Polypodum hydriforme
Porpita porpita
Rhodactis indosinensis
BIC
Number of components
E
V
TpG o/e
CpG o/e

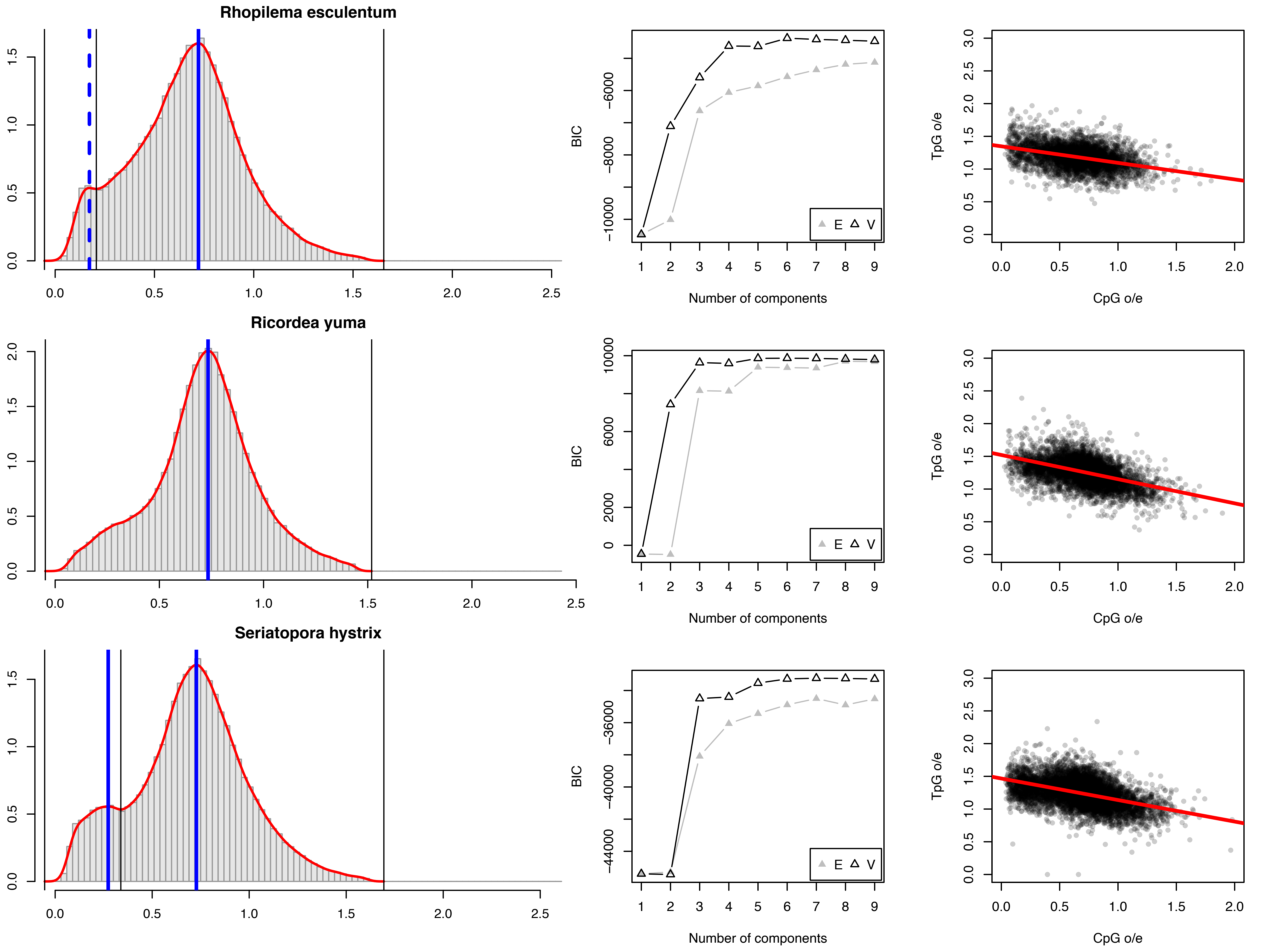
Rhopilema esculentum
Ricordea yuma
Seriatopora hystrix
BIC
Number of components
E
V
TpG o/e
CpG o/e

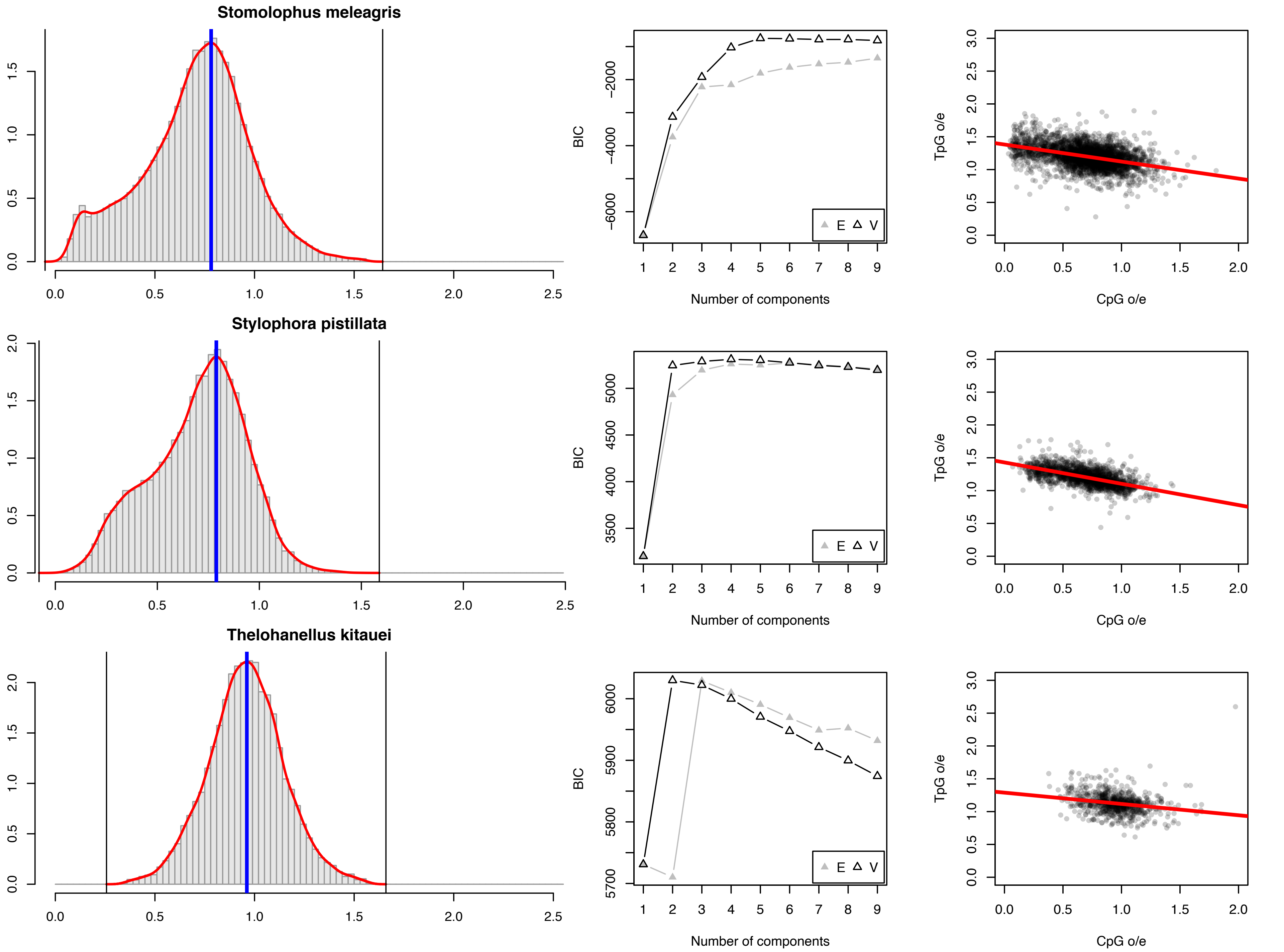

Stomolophus meleagris
Stylophora pistillata
Thelohanellus kitauei
BIC
Number of components
E
V
TpG o/e
CpG o/e

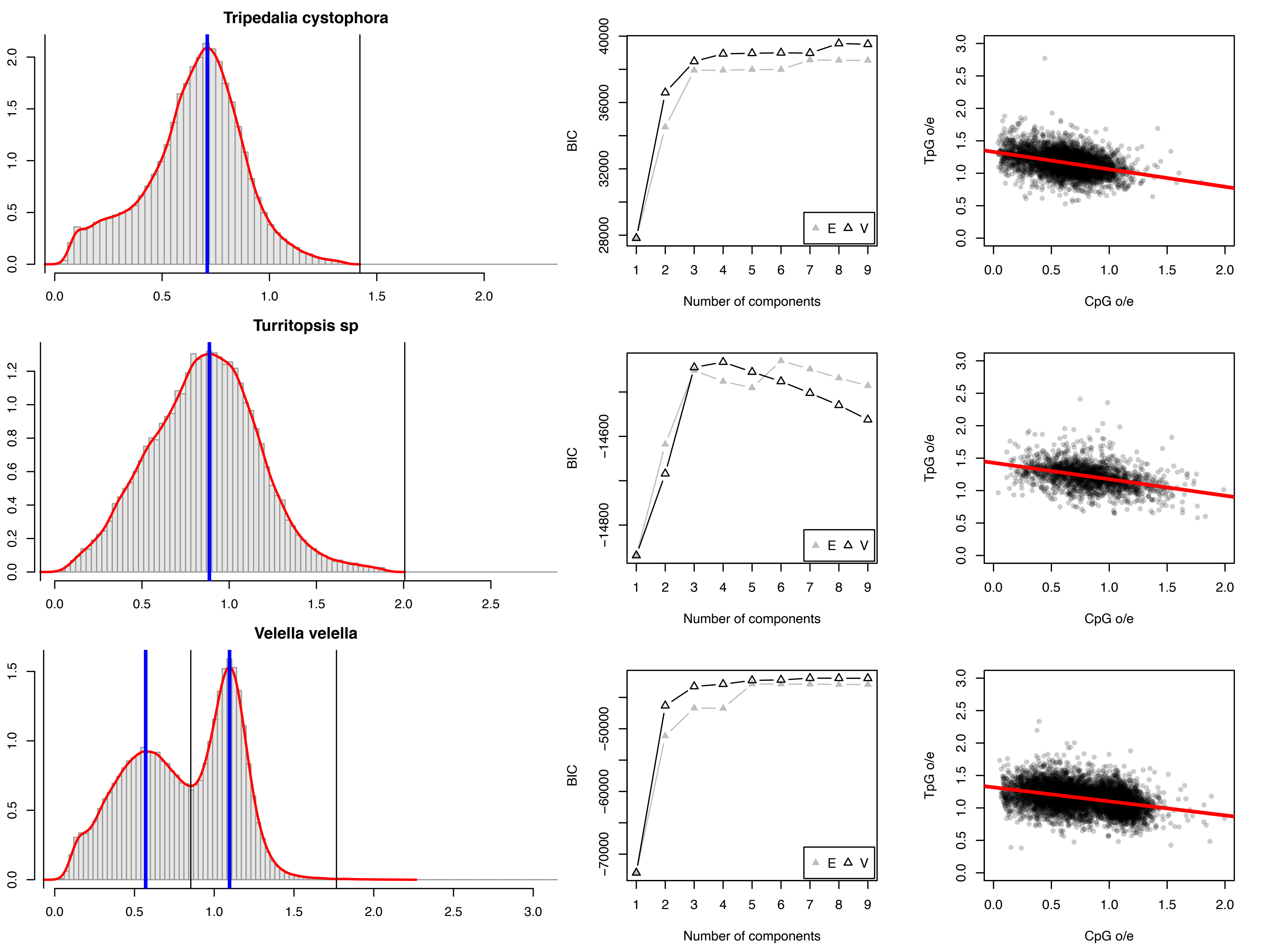
Tripedalia cystophora
Turritopsis sp
Velella velella
BIC
Number of components
E
V
TpG o/e
CpG o/e
40000
36000
32000
28000
−14600
−14800
−50000
−60000
−70000
0.0
0.5
1.0
1.5
2.0
2.5
3.0

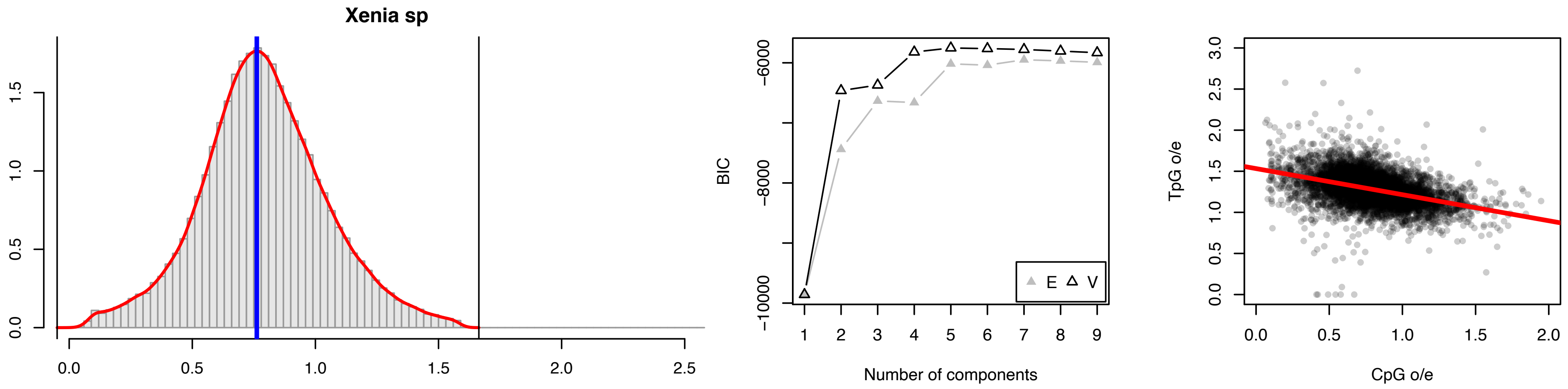

Xenia sp
BIC
Number of components
E
V
TpG o/e
CpG o/e
